# Contemporary disengagement from antiretroviral therapy in Khayelitsha, South Africa: A cohort study

**DOI:** 10.1371/journal.pmed.1002407

**Published:** 2017-11-07

**Authors:** Samantha R. Kaplan, Christa Oosthuizen, Kathryn Stinson, Francesca Little, Jonathan Euvrard, Michael Schomaker, Meg Osler, Katherine Hilderbrand, Andrew Boulle, Graeme Meintjes

**Affiliations:** 1 Yale School of Medicine, New Haven, Connecticut, United States of America; 2 Centre for Infectious Disease Epidemiology and Research, School of Public Health and Family Medicine, University of Cape Town, Cape Town, South Africa; 3 Médecins Sans Frontières (Southern Africa Medical Unit), Johannesburg, South Africa; 4 Department of Statistical Sciences, University of Cape Town, Cape Town, South Africa; 5 Institute of Infectious Disease and Molecular Medicine, University of Cape Town, Cape Town, South Africa; 6 Department of Health, Provincial Government of the Western Cape, Cape Town, South Africa; 7 Clinical Infectious Diseases Research Initiative, Institute of Infectious Disease and Molecular Medicine and Department of Medicine, University of Cape Town, Cape Town, South Africa; University of Southampton, UNITED KINGDOM

## Abstract

**Background:**

Retention in care is an essential component of meeting the UNAIDS “90-90-90” HIV treatment targets. In Khayelitsha township (population ~500,000) in Cape Town, South Africa, more than 50,000 patients have received antiretroviral therapy (ART) since the inception of this public-sector program in 2001. Disengagement from care remains an important challenge. We sought to determine the incidence of and risk factors associated with disengagement from care during 2013–2014 and outcomes for those who disengaged.

**Methods and findings:**

We conducted a retrospective cohort study of all patients ≥10 years of age who visited 1 of the 13 Khayelitsha ART clinics from 2013–2014 regardless of the date they initiated ART. We described the cumulative incidence of first disengagement (>180 days not attending clinic) between 1 January 2013 and 31 December 2014 using competing risks methods, enabling us to estimate disengagement incidence up to 10 years after ART initiation. We also described risk factors for disengagement based on a Cox proportional hazards model, using multiple imputation for missing data. We ascertained outcomes (death, return to care, hospital admission, other hospital contact, alive but not in care, no information) after disengagement until 30 June 2015 using province-wide health databases and the National Death Registry. Of 39,884 patients meeting our eligibility criteria, the median time on ART to 31 December 2014 was 33.6 months (IQR 12.4–63.2). Of the total study cohort, 592 (1.5%) died in the study period, 1,231 (3.1%) formally transferred out, 987 (2.5%) were silent transfers and visited another Western Cape province clinic within 180 days, 9,005 (22.6%) disengaged, and 28,069 (70.4%) remained in care. Cumulative incidence of disengagement from care was estimated to be 25.1% by 2 years and 50.3% by 5 years on ART. Key factors associated with disengagement (age, male sex, pregnancy at ART start [HR 1.58, 95% CI 1.47–1.69], most recent CD4 count) and retention (ART club membership, baseline CD4) after adjustment were similar to those found in previous studies; however, notably, the higher hazard of disengagement soon after starting ART was no longer present after adjusting for these risk factors. Of the 9,005 who disengaged, the 2 most common initial outcomes were return to ART care after 180 days (33%; *n* = 2,976) and being alive but not in care in the Western Cape (25%; *n* = 2,255). After disengagement, a total of 1,459 (16%) patients were hospitalized and 237 (3%) died. The median follow-up from date of disengagement to 30 June 2015 was 16.7 months (IQR 11–22.4). As we included only patient follow-up from 2013–2014 by design in order to maximize the generalizability of our findings to current programs, this limited our ability to more fully describe temporal trends in first disengagement.

**Conclusions:**

Twenty-three percent of ART patients in the large cohort of Khayelitsha, one of the oldest public-sector ART programs in South Africa, disengaged from care at least once in a contemporary 2-year period. Fifty-eight percent of these patients either subsequently returned to care (some “silently”) or remained alive without hospitalization, suggesting that many who are considered “lost” actually return to care, and that misclassification of “lost” patients is likely common in similar urban populations.

A challenge to meeting ART retention targets is developing, testing, and implementing program designs to target mobile populations and retain them in lifelong care. This should be guided by risk factors for disengagement and improving interlinkage of routine information systems to better support patient care across complex care platforms.

## Abstract 2

In a retrospective study, Samantha Kaplan and colleagues document the continuity of antiretroviral treatment and care in Khayelitsha, South Africa.

## Author summary

**Why was this study done?:**

- As of 2015, over 11 million people in sub-Saharan Africa were receiving antiretroviral therapy (ART) for treatment of HIV/AIDS, indicating the scale of patients who need lifelong treatment.
- An important component in controlling the HIV/AIDS epidemic is retaining patients in lifelong care. In addition to keeping individuals healthy, adherence to ART also helps curb the epidemic via viral suppression and prevention of onward transmission of the virus.
- The Khayelitsha ART program in Cape Town, South Africa is one of the oldest and largest public-sector ART programs in South Africa, and retention in care has been an ongoing issue needing attention.
- Previous studies have estimated rates of patients who are lost to follow-up (LTFU), but the program has grown substantially, and South African ART guidelines have changed repeatedly in recent years, supporting the need to reassess rates of disengagement in a larger and changing patient population.

**What did the researchers do and find?:**

- We conducted a cohort study using clinic database extracts of 39,884 patients ≥10 years of age who visited one of the 13 Khayelitsha ART clinics during 2013–2014.
- 9,005 (22.6%) patients disengaged from care (>180 days not attending clinic) at least once during 2013–2014, and an additional 987 (2.5%) silently transferred to another clinic in the same province. Using a statistical model, we estimated a cumulative incidence of disengagement of 25.1% at 2 years of study time.
- Factors associated with disengagement were age <30 years, male sex, and pregnancy at first ART visit. Factors associated with retention were baseline CD4 count <350 cells/μl and membership of an ART adherence club.
- In an analysis of outcomes for those who disengaged using electronic tracing methods, about a third of patients returned to care after disengagement, and an additional quarter were alive but not in care. During short-term follow-up, 3% of all patients who disengaged died.

**What do these findings mean?:**

- Rates of disengagement in this contemporary urban Khayelitsha cohort continue to be high; however, many patients return to care, sometimes at different clinics than the one in which they were originally initiated on ART. This suggests that many patients are potentially misclassified as lost to care, making program estimates for ART retention inaccurate, which may be more broadly applicable to similar urban sub-Saharan African ART cohorts.
- Additionally, our data suggest that systems need to account for and track mobile populations to retain them in care and prevent morbidity, mortality, and spread of HIV infection.

## Introduction

With the 2015 World Health Organization (WHO) guidelines recommending treatment for all HIV-infected individuals regardless of CD4 status and the continued high HIV incidence rates in endemic areas, there are increasing numbers of patients eligible for and starting lifelong antiretroviral therapy (ART). In order for health systems to meet the UNAIDS 90-90-90 treatment targets of patients receiving sustained ART and maintaining viral suppression, retention in care is an essential focus [1]. Viral suppression reduces HIV transmission [2], and in a modeling study has been shown to contribute to the public health goal of ending the HIV/AIDS epidemic [3]. Patients who disengage from care have an increased risk of poor health outcomes, transmitting HIV to others, and developing drug resistance, thereby undermining overall program impact as well as the global public health goal of ending the HIV epidemic. In Southern Africa, as of 2014, WHO-estimated ART retention rates after 5 years to be less than 50%, and the United States President’s Emergency Plan for AIDS Relief (PEPFAR) countries in this region reported 77% retention at 12 months in 2015 [4,5].

In Khayelitsha township (population approximately 500,000) in Cape Town, South Africa, an HIV treatment program was established in 2001 as a partnership between Médecins sans Frontières and the provincial government at 3 public-sector primary care clinics. This represented the first initiative to provide ART in the South African public sector [6]. As this program has matured and grown, disengagement from ART care has become a great challenge: cumulative loss to follow-up (LTFU) at 1 year was shown to have increased from 0% in 2001 to 7.6% in 2007, and a third of patients who were LTFU by 2008 were found to have died [7,8].

Over the 15 years since the Khayelitsha ART program’s inception, clinics have grown in size considerably [9], due to the high HIV prevalence and updating of the South African National ART guidelines, which have progressively expanded the eligibility criteria for ART initiation to higher CD4 count thresholds [10]. This has resulted in a massive increase of patients eligible for ART and an increase in the proportion of patients who are starting ART when asymptomatic. ART medication regimens have also become more tolerable, and fixed-dose combination formulations have improved convenience for patients. ART adherence clubs have been established for stable ART patients on treatment for >12 months who are virally suppressed. These clubs are managed by lay health workers and meet 5 times a year, allowing ease of medication refills and communal peer support [11,12]. These changes in the ART guidelines and service provision may alter the rates of disengagement reported previously [7,8]. In the current analysis, we merged different data sources to create a unique dataset of all Khayelitsha ART patients from provincial and municipal clinics. Using these routinely collected data, we sought to quantify disengagement from care and identify risk factors and outcomes for those patients who disengaged using electronic tracing methods.

## Methods

### Ethics statement

This study was approved by the Yale University Human Investigation Committee (Protocol # 1504015732) and the University of Cape Town Human Research Ethics Committee (HREC REF: 568/2015) per the protocol “Sub-study of protocol ‘Enhanced routine surveillance of patients in HIV care in Khayelitsha’ (HREC 395/2005)” (S1 Protocol Original). All data were extracted from databases containing patient data collected during routine ART or healthcare visits, and therefore per the approved protocol, informed consent was not obtained from individual patients. Patient data were de-identified prior to analysis. The original plan was to conduct a nested case-control study to determine risk factors for disengagement. However, early during data collection, the decision was taken to analyze risk factors on the whole cohort using Cox proportional hazards models rather than restrict to selected controls, as we determined that we would not be able to reliably abstract additional data on potential associations and confounders that were not already routinely available (S1 Protocol Amendment).

### Setting and inclusion criteria

A cohort study was conducted using data from all provincial and municipal public-sector ART clinics (*n* = 13) in Khayelitsha, Cape Town, South Africa. These clinics have provided ART to >50,000 patients since the program’s inception, with >30,000 patients receiving ART in 2015. The cohort is described in detail elsewhere [9]. Patients on ART in Khayelitsha constitute 17.5% of the total number on ART in the Western Cape province where treatment is provided across 250 clinics and roughly 1% of the national number of patients on ART across approximately 3,800 clinics.

**Fig 1:**
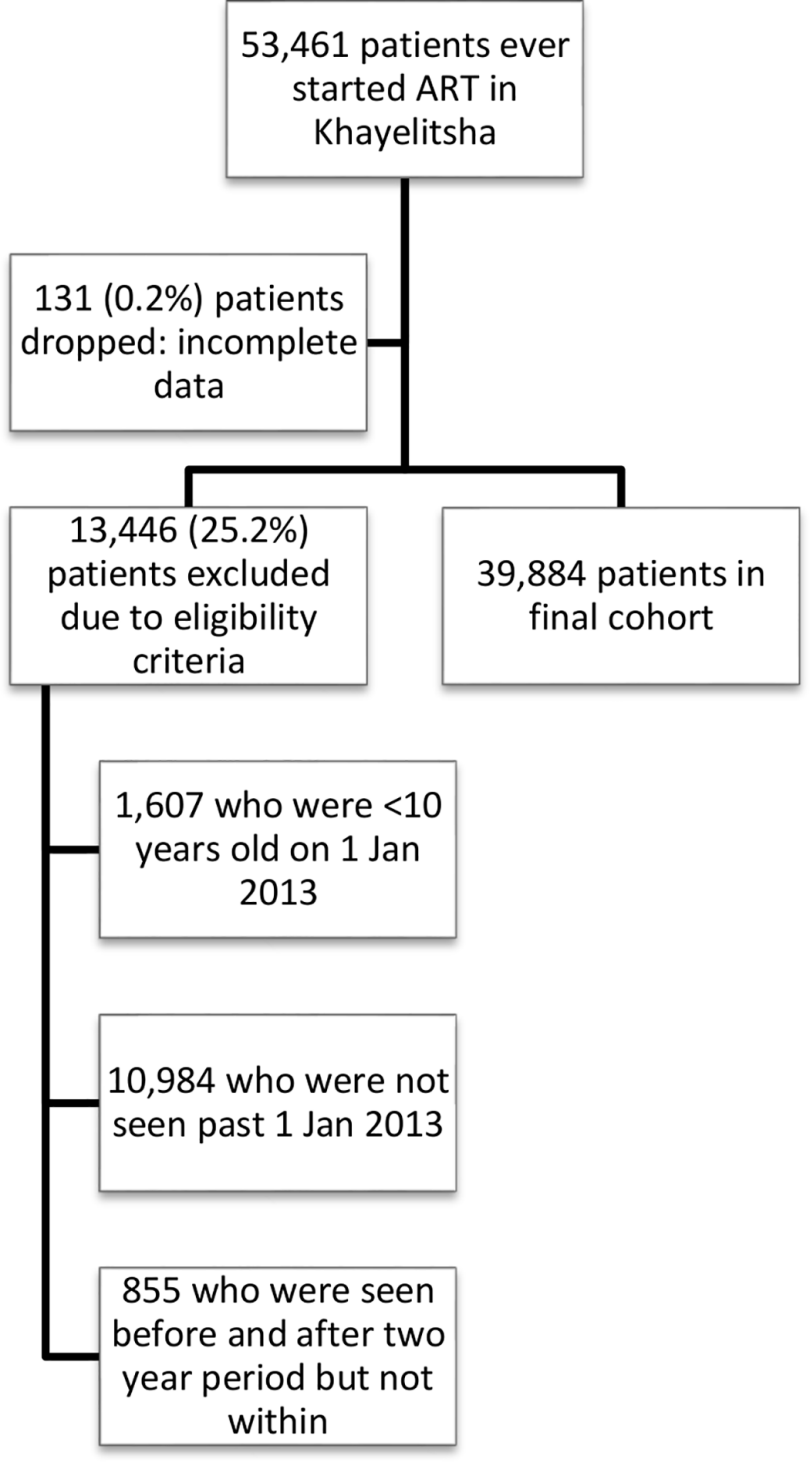
Flow diagram of study cohort. ART, antiretroviral therapy.

The study included all patients who had at least 1 visit at a Khayelitsha ART clinic between 1 January 2013 and 31 December 2014 regardless of the date they initiated ART, provided it was prior to 31 December 2014 (Fig 1). Patients not started on ART were excluded. This date range was selected to examine a contemporary cohort, but data included historical ART data prior to 2013 for patients selected for the cohort. Adults and adolescents age ≥10 years of age by 1 January 2013 were included, as we wanted to include adolescents in our analysis of risk factors for disengagement and age 10 is the start of adolescence as defined by WHO [13].

### ART eligibility, monitoring, and treatment regimens

ART eligibility criteria, patient monitoring, and treatment regimens have progressively changed since program initiation in 2001 (S1 Table). CD4 count and HIV viral load measurements were conducted at accredited National Health Laboratory Service (NHLS) laboratories.

### Data collection and management

In all Khayelitsha clinics, doctors and nurses who see patients enter visit data onto structured paper clinical records, which are subsequently captured on site into an electronic patient information system by data capturers. The municipal and provincial sites use different electronic patient information systems, but data are formatted and then exported by clinics based on an internationally implemented data exchange standard for HIV treatment data [14]. All record keeping and data capture are part of routine patient management, per provincial guidelines.

Civil identification numbers, when available, were used to ascertain or confirm dates of death up to 30 June 2015. Death dates were also ascertained from clinic records, if available. Western Cape province unique health identifiers were linked with the province-wide laboratory, pharmacy, and health facility visit databases to determine outcomes for those who disengaged, if available, up to 30 June 2015, and to supplement laboratory data where these were missing until 31 December 2014.

### Key definitions

Key study terms are defined below. Additional definitions can be found in S2 Table.

- *Disengagement*: a patient not seen in an ART clinic nor having an ART-related laboratory test or ART prescription in Khayelitsha for >180 days after his or her last visit date. This definition was chosen because patients may have up to 120-day intervals between scheduled visits and to align with previous studies [15,16]. It excludes “silent transfers.”
- *Date of disengagement*: start date of the 180-day period for which a patient was defined as disengaged.
- *Silent transfer*: patients who met the definition for disengagement but returned to an ART or primary care clinic somewhere else in the Western Cape province without formal transfer in ≤180 days.
- *Origin date*: The patient’s ART start date
- *Cohort entry date*: Either 1 January 2013 or the date of ART start or reentry into care if after 1 January 2013

### Data analysis

Data were analyzed using STATA/SE version 14.0 (StataCorp, College Station, TX, USA).

The cohort analysis was conducted in two parts: 1) analysis of time to disengagement from care in the cohort and analysis of risk factors for disengagement, using cumulative incidence curves and Cox proportional hazards models; and 2) for patients who disengaged from care, a description of outcomes after disengagement and times to these outcomes.

#### 1) Cumulative incidence estimates and Cox proportional hazards model for disengagement

**Fig 2:**
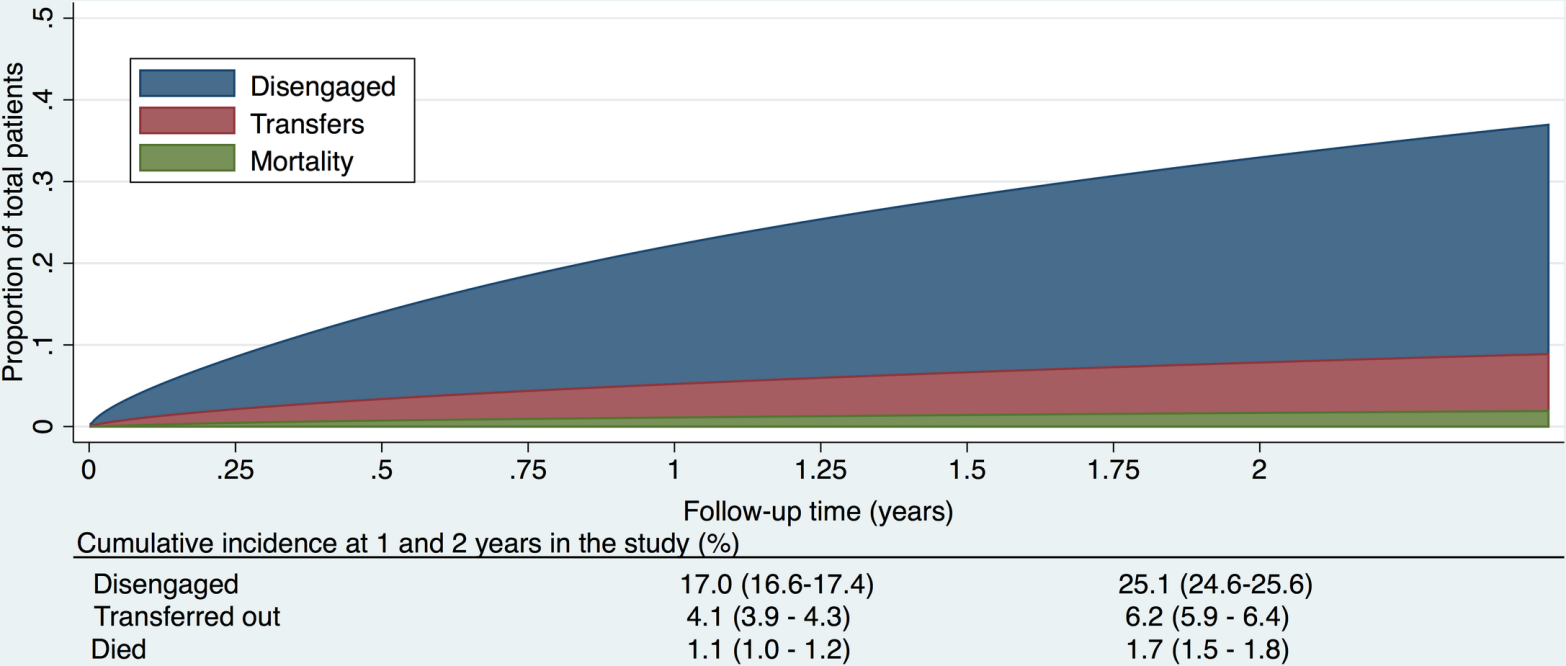
Cumulative incidence (competing risks analysis) of disengagement, transfer (including silent transfers), and mortality, as estimated by a flexible parametric survival model based on time to disengagement from cohort entry date to 31 December 2014.

**Fig 3:**
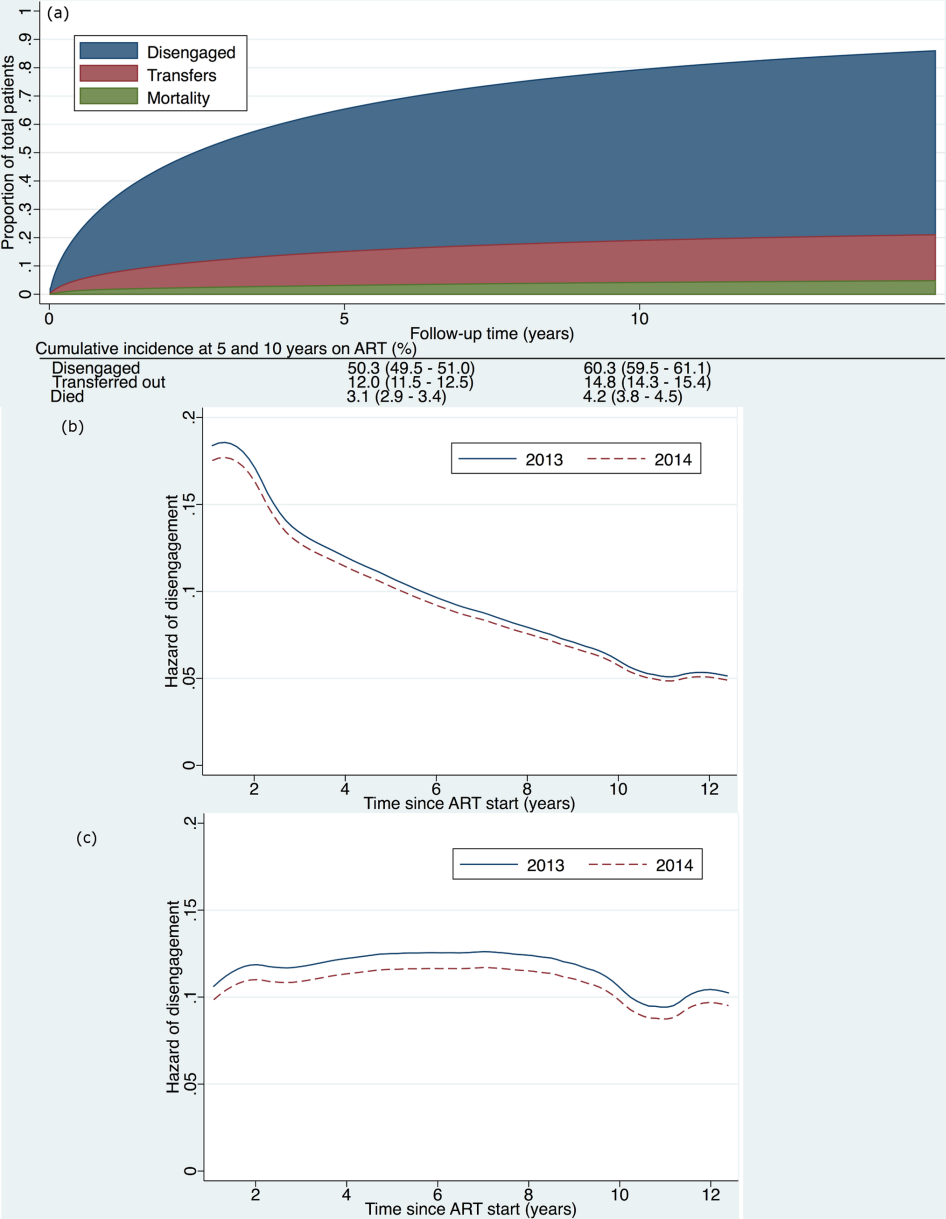
**(a) Cumulative incidence (competing risks analysis) of disengagement, transfer (including silent transfers), and mortality, as estimated by a flexible parametric survival model based on time contributed during a 2-year period, but analyzed relative to ART initiation date, allowing for delayed entry into the analysis. (b) Hazard of disengagement by duration since ART start, with calendar year (2013 versus 2014) fitted as a time-varying covariate in a Cox proportional hazards model. (c) Hazard of disengagement by duration since ART start, with calendar year (2013 versus 2014) fitted as a time-varying covariate in a Cox proportional hazards model adjusted for the patient characteristics listed in Table 2.** ART, antiretroviral therapy.

Entry into the study was at the beginning of 2013 for those who started ART prior to the analysis window, or at ART initiation if after 1 Jan 2013. Time to disengagement (failure) was defined as time from date of study entry to date of first disengagement within the window of 1 January 2013–31 December 2014 (Fig 2). Time origin was study entry and, in a second analysis, first ART start date, allowing for delayed entry into the survival analysis (Fig 3A). Date of disengagement was defined as the first date of the ≥180-day period that the patient was not in care. While survival time (until disengagement) was only calculated through 31 December 2014, the database was closed at 30 June 2015 to allow disengagement ascertainment (i.e., the opportunity to meet the disengagement definition) for those whose last visit was in late 2014. Therefore, if a patient disengaged prior to 1 January 2013, but returned to care prior to 1 January 2013, time to disengagement was calculated from their cohort entry date (or ART start date, depending on the analysis) until a date of disengagement within the cohort window if this occurred. Such a patient was also classified as “previously disengaged” at study entry. If a patient disengaged prior to 1 January 2013 but returned to care within the study window, the entry date was indicated as the date they reentered care within the study window and they were also classified as a patient who “previously disengaged.” For transferring patients (identified from clinical records, or by data clerks at the receiving clinics), if they transferred out prior to the study window but reentered within the study window, their entry date was their date of reentry but their origin date remained their ART start date. S1 Fig illustrates several examples of patients’ contribution to survival time. Possible outcomes for patients as of 31 December 2014 were 1) alive and in care; 2) dead; 3) transferred out; or 4) disengaged. The primary outcome was time to disengagement during the 2-year window of analysis. Secondary analyses focused on risk factors associated with disengagement.

For patients who officially transferred out within the study window, if they returned to care in Khayelitsha ≤180 days later, we ignored the transfer outcome and followed them until their next outcome (disengagement, transfer, death, or alive and in care). If the gap was >180 days (or they returned to care outside of Khayelitsha at any point), we classified them as transfer out and censored them at the transfer date. Silent transfers were censored on their last visit date in Khayelitsha. They were then reclassified as not disengaged. If their gap in care was >180 days, the patient remained classified as disengaged.

A patient was censored if they a) died (censored on death date); b) transferred (censored on transfer date); or c) were alive without disengagement at study end (administrative censor, on 31 December 2014). If a patient appeared in the National Death Registry within 90 days of a presumed date of disengagement, then they were reclassified to a death and not a patient who had disengaged. If a patient was seen in the cohort window for only 1 visit, we added 1 day to their outcome date so that they were included in the survival analysis [16].

*Descriptive analysis of cumulative incidence (competing risks analysis)*. Cumulative incidence curves were estimated for each of disengagement, transfer, and death, with the other events treated as competing events based on person time contributed during the analysis window, but analyzed from time since 1) entry into the study or 2) ART initiation. Death was a competing risk if it occurred ≤90 days after a date of disengagement or was the last outcome for a patient who had not disengaged prior to 31 December 2014. Transfer was a competing risk if it was the last outcome for a patient who had not disengaged prior to 31 December 2014, and included silent transfers. To estimate the hazard of disengagement over time, calendar time captured as year (2013 or 2014) was included as a time-varying covariate in the survival analysis. Follow-up time was split into 2 records for patients with follow-up time in both years. The underlying hazard was estimated following the fitting of the Cox models with and without adjustment for covariates.

*Model creation and testing*. Variables (decided upon a priori) were entered into a multivariable Cox proportional hazards model to determine risk factors for the hazard of disengagement. Cox proportional hazards assumptions (PHAs) were tested graphically for each variable. If the PHA was not met, the model was stratified with respect to the respective variable.

*Missing data*. We used imputation by chained equations [17] to impute missing data for TB, weight, club participation, CD4 count, HIV viral load, and drug regimens 5 times. The imputation model included all measured variables, used predictive mean matching for variables with skewed distributions (CD4 count, log HIV viral load), and used multinomial logistic regression for binary and categorical variables. The primary modeling results are based on the imputed data. In secondary analyses, we report results for a complete case analysis excluding missing data, as well as an imputed model restricted to patients with national identification numbers, which permitted linking to the National Death Registry.

#### 2) Disengagement outcome analysis

This analysis included only those who disengaged, and analyzed their subsequent outcomes after the first date of disengagement. For purposes of determining outcomes, the date of administrative censoring was extended to 30 June 2015. All outcomes after disengagement were ascertained from Western Cape province electronic data systems and the National Death Registry. Possible primary outcomes for those who disengaged were: 1) death; 2) return to care at a different facility within 180 days (silent transfer); 3) return to care after 180 days; 4) hospital admission; 5) other hospital contact (outpatient or emergency visit); 6) alive on 30 June 2015 if they had a national identification number but no death date was found in the National Death Registry; or 7) no information, still disengaged. Primary outcomes were identified as the first of these outcomes after the date of disengagement. Secondary outcomes were time to return to care and time to death after disengagement. Overall deaths and hospitalizations at any point post disengagement until 30 June 2015 were also calculated. We conducted a sensitivity analysis of disengagement outcomes for those with national identification numbers to restrict analyses to only those with reliable vital status ascertainment.

*Geographic Information System (GIS) analysis*. GIS mapping and analysis were conducted using ArcGIS Online based on official facility coordinates in order to map the locations at which silent transfers and those who disengaged returned to care.

## Results

### Cohort characteristics

A total of 53,461 patients initiated ART at any Khayelitsha site since program inception through 31 December 2014. For the cohort study, we excluded 11,839 patients who did not have a visit in the time period between 1 January 2013 and 31 December 2014, and 1,607 who were less than 10 years old at 1 January 2013. An additional 131 were excluded due to incomplete data (Fig 1). Of the 39,884 patients remaining (Table 1), the median follow-up from ART start date to 31 December 2014 was 33.6 months (IQR 12.4–63.2). A total of 25,864 patients started ART prior to 1 January 2013 (contrasted with those who started after this date in S3 Table). We also indicate the proportion of patients with missing data for each variable (Table 1, S3 Table).

**Table 1:** Characteristics of patients who did and did not disengage from care.

Variable	Patients who did not disengage			Patients who disengaged (>180 days)			Whole cohort		
# of participants (*n*)	30,879	# of patients with complete data	% complete data	9,005	# of patients with complete data	% complete data	39,884	# of patients with complete data	% complete data
Age at 1 January 2013, years (median, IQR)	35.2 (29.2–41.6)	30,879	100.0%	32.0 (26.6–39.0)	9,005	100.0%	34.4 (28.5–41.0)	39,884	100.0%
Male sex (*n*, %)	8,862 (28.7%)	30,879	100.0%	2,825 (31.4%)	9,005	100.0%	11,687 (29.3%)	39,884	100.0%
Months on ART at 31 December 2014 (median, IQR)	38.3 (16.8–68.4)	30,879	100.0%	15.8 (4.6–41)	9,005	100.0%	33.6 (12.4–63.2)	39,884	100.0%
Median year of starting ART (median, IQR)	2011 (2009–2013)	30,879	100.0%	2012 (2010–2013)	9,005	100.0%	2012 (2009–2013)	39,884	100.0%
Baseline CD4 count, cells/μL (median, IQR)	185 (101–274)	27,398	88.7%	200 (114–299)	7,815	86.8%	188 (104–280)	35,213	88.3%
Baseline CD4 by category (cells/μL; *n*, %)	>350: 2,703 (8.8%)			>350: 1,164 (12.9%)	9,005	100.0%	>350: 3,867 (9.7%)	39,884	100.0%
	200–350: 9,659 (31.3%)			200–350: 2,770 (30.8%)			200–350: 12,429 (31.2%)		
	50–200: 11,835 (38.3%)			50–200: 3,076 (34.2%)			50–200: 14,911 (37.4%)		
	<50: 3,201 (10.4%)			<50: 805 (8.9%)			<50: 4,006 (10.0%)		
	missing: 3,481 (11.3%)			missing: 1,190 (13.2%)			missing: 4,671 (11.7%)		
Most recent CD4 count as of 31 Dec 2014, cells/**μ**L (median, IQR)	443 (284–616)	26,559	86.0%	325 (195–495)	7,936	88.1%	415 (259–593)	34,495	86.5%
Most recent viral load >1,000 on ART as of 31 Dec 2014 (*n*, %)	1,812 (10.5%)	17,191	55.7%	1,183 (23.4%)	5,060	56.2%	2,995 (13.5%)	22,251	55.8%
Achieved viral suppression on ART (<400) (n, %)	22,669 (95.6%)	23,734	76.9%	4,513 (85.9%)	5,252	58.3%	27,212 (93.9%)	28,986	72.7%
Initiated ART during pregnancy (women only) (*n*, %)	2,554 (11.7%)	21,911	99.5%	1,231 (20.0%)	6,150	99.5%	3,785 (13.5%)	28,061	99.5%
TB treatment at ART initiation (*n*, %)	6,612 (21.5%)	30,721	99.5%	1,981 (22.1%)	8,962	99.5%	8,593 (21.7%)	39,683	99.5%
Ever transferred within Khayelitsha (*n*, %)	1,315 (4.3%)	30,879	100.0%	371 (4.1%)	9,005	100.0%	1,686 (4.2%)	39,884	100.0%
Transferred into ART care (*n*, %)	3,401 (11.0%)	30,879	100.0%	1042 (11.6%)	9,005	100.0%	4,443 (11.1%)	39,884	100.0%
ART club membership, ever (*n*, %) *	6,000 (30.1%)	19,957	64.6%	409 (7.2%)	5,703	63.3%	6,409 (25.0%)	25,660	64.3%
Weight at ART baseline (median, IQR) *	64 (55.6–75)	18,589	60.2%	63 (55–74)	5,197	57.7%	64 (55.5–74.9)	23,786	59.6%
Baseline ART regimen drug 1 (*n*, %)	TDF 18,189 (58.9%)	30,879	100.0%	TDF 6,268 (69.6%)	9,005	100.0%	TDF 24,457 (61.3%)	39,884	100.0%
	d4T 7,322 (23.7%)			d4T 1,424 (15.8%)			d4T 8,746 (21.9%)		
	AZT 3,116 (10.1%)			AZT 661 (7.4%)			AZT 3,777 (9.5%)		
	ABC 95 (0.3%)			ABC 24 (0.3%)			ABC 119 (0.3%)		
	missing 2,157 (7.0%)			missing 628 (7.0%)			missing 2,785 (7.0%)		
Baseline ART regimen drug 3 (*n*, %)	EFV 22,815 (73.9%)	30,879	100.0%	EFV 7,157 (79.5%))	9,005	100.0%	EFV 29,972 (75.2%)	39,884	100.0%
	NVP 5,590 (18.1%)			NVP 1,108 (12.3%)			NVP 6,698 (16.8%)		
	LPV/r 200 (0.7%)			LPV/r 56 (0.6%)			LPV/r 256 (0.6%)		
	Other 15 (0.05%)			Other 5 (0.06%)			other 20 (0.05%)		
	missing 2,259 (7.3%)			missing 679 (7.5%)			missing 2,938 (7.4%)		
Most recent ART regimen drug 1 as of 31 Dec 2014 (*n*, %)	TDF 24,646 (79.8%)	30,879	100.0%	TDF 7,406 (82.2%)	9,005	100.0%	TDF 32,052 (80.4%)	39,884	100.0%
	AZT 4,258 (13.8%)			AZT 916 (10.2%)			AZT 5,174 (13.0%)		
	d4T 1,386 (4.5%)			d4T 538 (6.0%)			d4T 1,924 (4.8%)		
	ABC 74 (0.2%)			ABC 18 (0.2%)			ABC 92 (0.2%)		
	missing 515 (1.7%)			missing 127 (1.4%)			missing 642 (1.6%)		
Most recent ART regimen drug 3 as of 31 Dec 2014 (*n*, %)	EFV 24,706 (80.0%)	30,879	100.0%	EFV 7,542 (83.8%)	9,005	100.0%	EFV 32,248 (80.9%)	39,884	100.0%
	NVP 2,933 (9.5%)			NVP 667 (7.4%)			NVP 3,600 (9.0%)		
	LPV/r 2,783 (9.0%)			LPV/r 687 (7.7%)			LPV/r 3,470 (8.7%)		
	Other 116 (0.4%)			Other 10 (0.1%)			other 126 (0.3%)		
	missing 341 (1.1%)			missing 99 (1.1%)			missing 440 (1.1%)		
Previous gap in care of >180 days (*n*, %)	3,677 (11.9%)	30,879	100.0%	1,737 (19.3%)	9,005	100.0%	5,414 (13.6%)	39,884	100.0%

*Variables/data available at provincial clinics only

ABC, abacavir; ART, antiretroviral therapy; AZT, zidovudine; d4T, stavudine; EFV, efavirenz; LPV/r, lopinavir/ritonavir; NVP, nevirapine; TB, tuberculosis; TDF, tenofovir.

### Disengagement from care and other outcomes during the two-year study period

From the perspective of the Khayelitsha clinic system, a total of 9,992 (25.1%) patients disengaged from care. However, after linkage of these patients to Western Cape data systems, we found that patients who disengaged (excluding those who silently transferred) numbered 9,005 (22.6%). From this point on in the manuscript, “patients who disengaged” refers to those who disengaged, excluding silent transfers.

As of 31 December 2014, of the total cohort, 592 (1.5%) died, 1,231 (3.1%) transferred out, 987 (2.5%) were silent transfers and visited another ART or primary care clinic in the same province (Western Cape) within 180 days of their last visit date, 9,005 (22.6%) disengaged, and 28,069 (70.4%) were in care. Of those in our study cohort, 1,179 (3.0%) patients disengaged prior to 2013 but returned to care prior to 1 January 2013. 4,156 (10.4%) disengaged prior to 2013 but returned within the study window.

Of the patients who disengaged, 5,463 (60.7%) had South African national identification numbers and could be linked to the National Death Registry. Total mortality for the entire cohort as of 30 June 2015 was 2.4% (*n* = 939), and 3.9% (*n* = 822) when restricted to those with national identification numbers.

The cumulative incidence of disengagement from care, before any other event could occur, was estimated to be 25.1% at 2 years, analyzed by time in the study (Fig 2). The cumulative incidence of disengagement was 50.3% and 60.3% at 5 and 10 years on ART, respectively (Fig 3A), based on the person time contributed during the analysis window but analyzed relative to ART initiation date. The higher hazard of disengagement soon after starting ART was largely attenuated after adjusting for the patient characteristics included in Table 2, without evidence of a temporal effect comparing 2013 to 2014 (Fig 3B and 3C).

### Factors associated with disengagement

The strongest adjusted associations with disengagement were most recent CD4 count <350 cells/μl (CD4 200–350 hazard ratio (HR) 2.03; 95% CI 1.91–2.15; CD4 50–200 HR 3.07; 95% CI 2.84–3.31; CD4 <50 HR 3.34; 95% CI 2.92–3.83, all relative to CD4 > 350), use of d4T (stavudine) at last visit (HR 1.72; 95% CI 1.57–1.89), and pregnancy at ART start (HR 1.58; 95% CI 1.47–1.69) (Table 2).

**Table 2:** Cox proportional hazards model for risk of disengagement, univariate and multivariable, imputed.

	Univariate: IMPUTED (*n* = 39,884)		Multivariable: IMPUTED* (*n* = 39,884)	
Variable	HR	95% CI	HR	95% CI
**Age category**				
*10–20 years*	1.58	1.42–1.75	1.38	1.24–1.54
*20–30 yrs*	1.50	1.42–1.57	1.46	1.38–1.54
*30–40 yrs*	ref	ref	ref	ref
*40–50 yrs*	0.87	0.82–0.93	0.90	0.85–0.96
*50–60 yrs*	0.90	0.82–1.00	0.91	0.82–1.01
*>60 yrs*	1.24	1.03–1.50	1.08	0.89–1.31
**Sex/pregnancy**				
*Nonpregnant women*	ref	ref	ref	ref
*Pregnant at ART initiation (women)*	1.73	1.62–1.84	1.58	1.47–1.69
*Men*	1.22	1.17–1.28	1.14	1.08–1.20
**Year of ART initiation**	1.17	1.13–1.21	-	-
**TB treatment at ART initiation**	1.06	1.01–1.19	strata	strata
**Transfer into care**	1.10	1.04–1.18	-	-
**Transfer in Khayelitsha**	1.10	0.99–1.22	-	-
**Any transfer**	1.13	1.07–1.20	strata	strata
**Previous gap in care of >180 days**	2.80	2.65–2.97	strata	strata
**Provincial clinic**	1.06	1.02–1.11	1.06	1.01–1.12
**Baseline CD4 (cells/μl)**				
*>350*	ref	ref	ref	ref
*200–350*	0.71	0.67–0.76	0.60	0.56–0.65
*50–200*	0.77	0.71–0.82	0.46	0.43–0.50
*<50*	0.78	0.72–0.86	0.39	0.35–0.44
**Most recent CD4 as of 31 Dec 2014 (cells/μl)**				
*>350*	ref	ref	ref	ref
*200–350*	1.91	1.81–2.02	2.03	1.91–2.15
*50–200*	2.89	2.72–3.07	3.07	2.84–3.31
*<50*	3.14	2.79–3.52	3.34	2.92–3.83
**Maximum viral load**				
*<1*,*000*	ref	ref	-	-
*1*,*000–10*,*000*	1.48	1.35–1.61	-	-
*10*,*000–100*,*000*	1.68	1.56–1.80	-	-
*>100*,*000*	1.71	1.59–1.83	-	-
**Most recent viral load as of 31 Dec 2014**	** **	** **	** **	** **
*<1*,*000*	ref	ref	-	-
*1*,*000–10*,*000*	1.98	1.76–2.23	-	-
*10*,*000–100*,*000*	2.54	2.34–2.77	-	-
*>100*,*000*	2.87	2.60–3.18	-	-
**Viral load suppressed ever during ART**	0.38	0.36–0.41	0.58	0.53–0.64
**ART adherence club membership, ever**	0.23	0.21–0.25	0.29	0.26–0.32
**Weight at ART initiation (*n* = 23,786)**	0.99	0.99–1.00	-	-
**Baseline ART regimen drug 1**				
*TDF*	ref	ref	-	-
*AZT*	0.95	0.86–1.06	-	-
*ABC*	0.81	0.86–1.06	-	-
*d4T*	0.96	0.87–1.06	-	-
**Baseline ART regimen drug 3**				
*EFV*	ref	ref	-	-
*NVP*	0.92	0.85–0.99	-	-
*LPV/r*	1.03	0.80–1.33	-	-
*Other*	1.33	0.53–3.33	-	-
**Most recent ART regimen drug 1 as of 31 Dec 2014**	** **	** **	** **	** **
*Other*	ref	ref	ref	ref
*d4T*	1.81	1.66–1.98	1.72	1.57–1.89
**Most recent ART regimen drug 3 as of 31 Dec 2014**	** **	** **	** **	** **
*EFV*	ref	ref	ref	ref
*NVP*	1.04	0.96–1.14	1.17	1.08–1.28
*LPV/r*	1.03	0.95–1.12	0.65	0.60–0.71
*Other*	0.39	0.21–0.72	0.21	0.11–0.39

ABC, abacavir; ART, antiretroviral therapy; AZT, zidovudine; d4T, stavudine; EFV, efavirenz; HR, hazard ratio; LPV/r, lopinavir/ritonavir; NVP, nevirapine; ref, reference; TB, tuberculosis; TDF, tenofovir; VL, viral load; yrs, years

*All variables with estimates listed were included in the multivariable model.

Other associations included younger age group (<30 years) and male sex. The factors associated with retention were ART adherence club membership (HR 0.29; 95% CI 0.26–0.32), a suppressed HIV viral load at any point during ART (HR 0.58; 95% CI 0.53–0.64), and a baseline CD4 count <350 cells/μl (CD4 200–350 HR 0.60; 95% CI 0.56–0.65; CD4 50–200 HR 0.46; 95% CI 0.43–0.50; CD4<50 HR 0.39; 95% CI 0.35–0.44, all relative to CD4 > 350) (Table 2). Post-imputation distributions for variables with skewed distributions were compared with observed distributions and were acceptable (S2 Fig). Proportional hazard assumptions for imputed variables were also met, with only a small deviation for long follow-up for ART regimen drug 3 (S3 Fig). Sensitivity analyses restricted to complete data or to patients with national identification numbers did not materially alter these associations (S4 Table).

### Outcomes after disengagement

Of those who disengaged (*n* = 9,005), the median length of follow-up from date of disengagement to 30 June 2015 was 16.7 months (IQR 11–22.4). 7,061 (78.4%) of those who disengaged could be linked using a national identification number or medical record number for outcomes analysis. The 2 most common first outcomes were return to ART care after 180 days (33%; *n* = 2,976), followed by alive but not in care (25%; *n* = 2,255; valid national identification number allowing ascertainment of vital status, but no other information) (Table 3). Because only 60.7% (*n* = 5,463) of those who disengaged had valid national identification numbers that could be linked to the National Death Registry, we conducted a sensitivity analysis of only these patients (Table 3). In this analysis, 75% (*n* = 4,125) had either returned to care (34%; *n* = 1,877) or remained disengaged but were known to be alive (41%; *n* = 2,248), with the remainder having been admitted or seen as an outpatient or emergency visit at a hospital (24%; *n* = 1,280) or having died (1%; *n* = 58) as their first outcome. Not restricting to the first outcome, 3% (*n* = 237) of those who disengaged died at any point during subsequent follow-up, and 16% (*n* = 1,459) were admitted to the hospital (S5 Table).

**Table 3:** Initial outcomes for patients who disengaged, as ascertained from Western Cape province data systems until 30 June 2015.

	Patients who disengaged + silent transfers (*n* = 9,992)	All patients who disengaged (*n* = 9,005)	Patients who disengaged with identification numbers, allowing accurate mortality ascertainment (*n* = 5,463)
**Return to care**	2,976 (30%)	2,976 (33%)	1,877 (34%)
Alive as of 30 June 2015 (have ID #s)*	2,255 (23%)	2,255 (25%)	2,248 (41%)
**No information**	1,944 (19%)	1,944 (22%)	-
**Other hospital contact**	1,218 (12%)	1,218 (13%)	896 (17%)
**Silent transfer**	987 (10%)	-	-
**Hospital admission**	545 (5%)	545 (6%)	384 (7%)
**Death**	67 (1%)	67 (1%)	58 (1%)

*Alive as of 30 June 2015 refers to patients who had valid national identification numbers but were not found in care anywhere in the Western Cape nor were they found to be dead. Therefore, “alive” is the only outcome we could ascertain.

**Fig 4:**
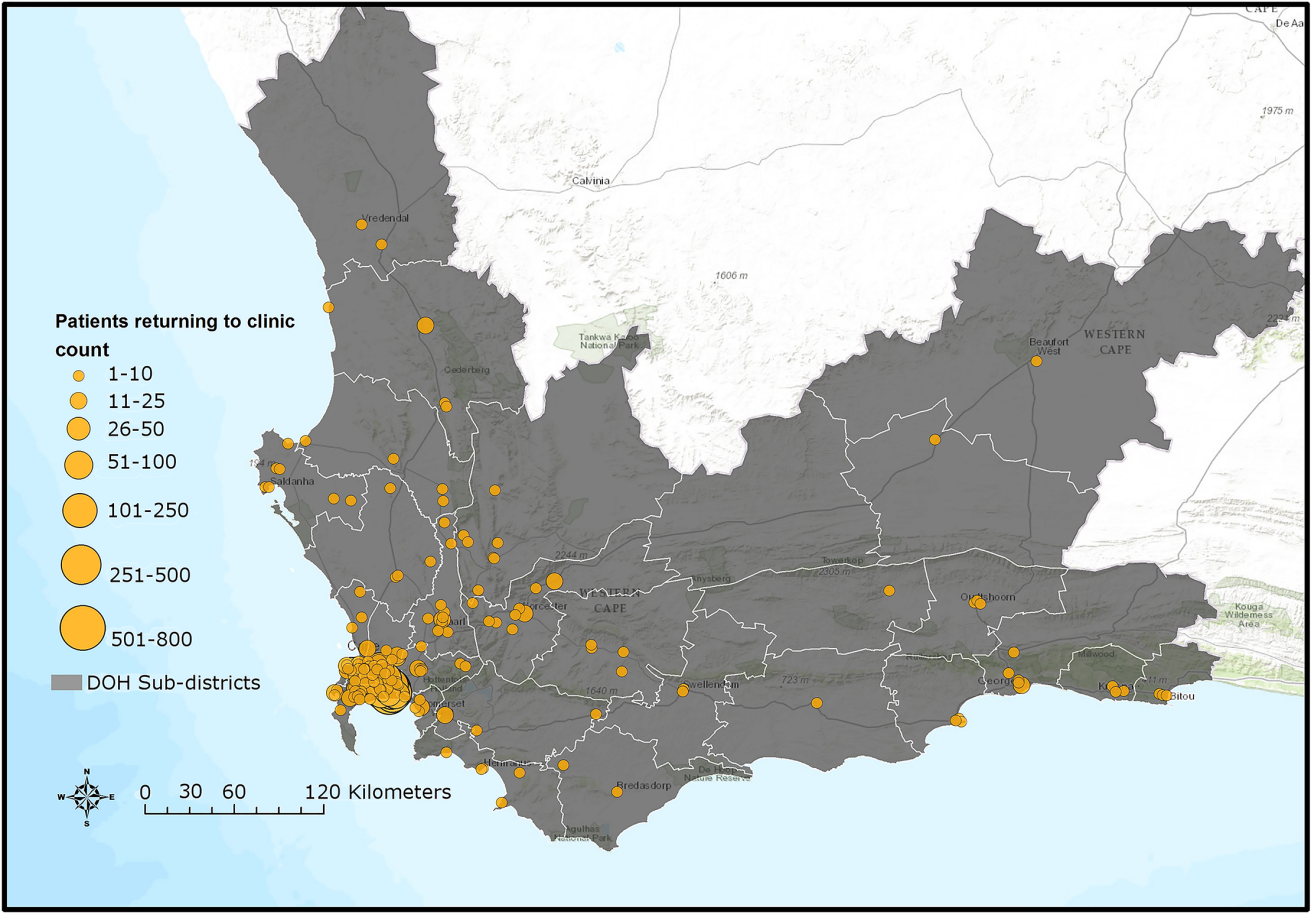
Map of Western Cape province indicating clinics where silent transfers and patients who disengaged returned to care until 30 June 2015. DOH, Department of Health. **Sources:** Basemap: Used with permission. Copyright 2017 Esri, ArcGIS Desktop, Western Cape Department of Health, and the GIS User Community. CC BY 4.0. South African Department of Health Layer: Boundaries of the Department of Health Sub-districts falling under the authority of the Western Cape Government and the City of Cape Town municipality (data provided June 2016 by Deputy Director, Information Management Health, Western Cape Government, South Africa). South African public health facilities layer: Locations of facilities falling under the authority of the Western Cape Government and the City of Cape Town. Includes some Inactive facilities as listed in the operational Sinjani system (data provided June 2016 by Deputy Director, Information Management Health, Western Cape Government, South Africa). Software: ESRI 2016. ArcGIS Desktop: version 10.4. Redlands, CA: Environmental Systems Research Institute.

A Kaplan-Meier analysis of time to returning to care for patients who disengaged estimated that approximately 50% of patients who disengage will return to care by 2.5 years (S4A Fig). A Kaplan-Meier analysis for time to death after disengagement, restricted to patients with valid identification numbers, estimated that >90% of patients were alive at 2.5 years post-disengagement (S4B Fig). For those patients who silently transferred or disengaged and then returned to care >180 days later, GIS mapping indicated that most of these patients returned to care very close to Khayelitsha (some at the same clinic), but many also returned to a variety of locations throughout the Western Cape province (Fig 4). Median distance from Khayelitsha to clinic of return was 3.8 km (IQR 2.7–9.6, range 0.3–434), an estimate that includes patients who returned to the same clinic after 180 days. Seven percent (*n* = 207) returned >50km from Khayelitsha, but within the Western Cape province. In a logistic regression model, some of the associations with failure to re-engage in care after disengagement were the same as those associated with disengagement: male sex (OR 1.16; 95% CI 1.04–1.29) and pregnancy at ART start (OR 1.36; 95% CI 1.15–1.61). Older age was associated with failure to return (>60 years; OR 1.76; 95% CI 1.11–2.78). However, younger age and CD4 count were not associated with failure to return to care (S6 and S7 Tables).

## Discussion

In this study, we examined disengagement from ART care during 2013–2014 among patients of the large, peri-urban cohort in Khayelitsha—one of the oldest public-sector ART cohorts in South Africa. Roughly 1 in 5 patients disengaged from care, demonstrating a high rate of disengagement and a key challenge to reaching the UNAIDS 90-90-90 treatment targets. Factors associated with disengagement were age <30 years, male sex, pregnancy at ART initiation, and last CD4 count <350 cells/μl. Factors associated with retention were ART adherence club membership and baseline CD4 <350 cells/μl. However, despite the high incidence of disengagement, many of those who disengaged did not do so permanently. While 48% (*n* = 4,199) of patients could not be traced (either did not have a national identification number or had an ID number and/or medical record number but no additional data were found), and 16% (*n* = 1,459) were admitted to the hospital at some point after disengagement, roughly 1 in 3 patients returned to care within the province during the study period, and half were estimated to return to care within 2.5 years. Additionally, not included in the overall estimate of disengagement are the 2.5% of silent transfers who appeared to disengage from a clinic perspective but were actually in care elsewhere in the Western Cape province when province-wide data linkage was performed. These data indicate that a substantial proportion of patients are cycling in and out of care as well as transferring elsewhere in the province (often “silently”), and potentially to facilities outside of the Western Cape province (something that our study could not ascertain).

### Disengagement in Khayelitsha

Previous studies of the Khayelitsha cohort have reported on disengagement (termed “lost to follow-up, LTFU”, in those studies) and mortality up until 2009 [7,8,18]. These studies are not directly comparable, as they calculated LTFU at the end of a defined period allowing for prior return to care, as opposed to the current study, in which we defined disengagement as the first episode in a 2-year period. In doing so, we report a contemporary cross-sectional perspective of disengagement rather than a cumulative snapshot. Temporal trends based on the former approach have further been shown to be biased [19]. Further, the current study accounted for silent transfers and mortality through data linkage, whereas the former studies only accounted for mortality. Nevertheless, the most recent of the previous studies estimated cumulative LTFU of 23% at 5 years on ART in 2008 [8], compared to the current contemporary estimate of 25% meeting the definition of LTFU at least once in a 2-year period, irrespective of duration on ART or previous disengagement. When extrapolated, and assuming that this incidence is unchanged over time and is homogeneously distributed, this would mean that 50% of patients would meet this definition by 5 years on ART. In spite of improved ascertainment of patients transferring their care, the study provides continued and robust evidence of high rates of disengagement, underscoring the importance of interventions targeted primarily at retention in continuous ART care across a platform or jurisdiction.

### Risk factors for disengagement

Our assessment of risk factors associated with disengagement was limited to those in the Khayelitsha database. Male sex and pregnancy at ART initiation were strongly associated with disengagement, consistent with previous studies [7,20–25]. While d4T—with its high risk for drug toxicity [26,27]—was associated with increased risk of disengagement in our study, the effect is potentially confounded, as d4T was only prescribed earlier in the cohort period. Nonetheless, associations with ART toxicity and disengagement have been reported, indicating that ART drugs that minimize toxicities may decrease the risk of disengagement [28].

Our results indicate that a baseline CD4 count <350 cells/μl was associated with retention, and that a most recent CD4 count <350 cells/μl was predictive of disengagement. While these findings could suggest that those who are less sick early in treatment are more likely to disengage in line with previous studies [22,29,30], this might be confounded by non-CD4-based clinical indications for ART initiation, as clinical guidelines at the time did not provide for universal eligibility of patients with CD4 counts in this range.

We also found an association with retention for adherence club membership in Khayelitsha—which is confounded by indication—as more stable, engaged patients (on ART for >12 months, virally suppressed) are referred to adherence clubs. Twenty-six percent of patients in the entire cohort were in clubs by June 2014, and recent data have shown that adherence club membership is associated with a 67% reduction in LTFU [9,31].

We found that the higher hazard of disengagement soon after starting ART seemed to be accounted for by other risk factors in that this was no longer present in adjusted analyses (Fig 3C). Other studies have suggested that there is a higher risk of disengagement early in ART treatment [24,32], but they did not adjust for other risk factors as we did in our analysis. This lends support to the conclusion that the high early loss to ART care may be accounted for by key demographic and clinical patient characteristics such as age, sex, pregnancy, and CD4 count.

### Silent transfers and return to care: Cyclical engagement

The combination of high proportions of patients being silent transfers or returning to care after disengagement in this setting casts the challenge as cycling in and out of care and between facilities, rather than as definitive losses to care. This represents a major shift in the way ART care is now being delivered. Many of these patients spent substantial time out of care and were likely viremic. This cyclical engagement undermines the ultimate goals of the UNAIDS 90-90-90 agenda because during these interruptions in care, HIV may progress in individuals, and transmission of HIV to others is more likely.

In the current analysis, the 10% of disengagements that were in fact silent transfers underscore the relative importance of undocumented transfer being a reason for apparent disengagement. While the estimate may sound lower than previous findings of this figure, which is closer to 20% in the Western Cape [33] and globally [34], we required that the patient transfer be to a site outside of Khayelitsha in order to exclude transfers within the subdistrict and that the transfer be within 6 months of the last visit.

In terms of return to care, one study in the Cape Town area found that 33% of those who disengaged returned to care with the probability of resuming within 3 years of 42%, a median of 228 days after disengagement [22], very similar to the findings in the current study. In terms of mobility issues, a rural South African cohort study found that 32% of those LTFU had migrated outside of the area after disengagement [29]. Indeed, data from Khayelitsha and the neighboring Mitchell’s Plain district have indicated that approximately 84% of black South African adults were born outside of Cape Town, the majority in the Eastern Cape, and these people frequently travel to visit their families in that province or travel between the provinces for employment or annual vacation [35–37].

However, half of patients who disengaged in this study did not return to care in the Western Cape province. Over 15% of the patients in this study were admitted to a hospital after disengagement, and 3% died. These adverse events could illustrate the potential clinical consequences of disengagement with associated healthcare costs. Other studies have illustrated such problems that arise when patients disengage from ART care: one study indicated that HIV contributed to over 60% of medical admissions to a South African district hospital in Cape Town from 2012–2013, and 19.3% of these HIV-infected patients had interrupted ART therapy [38]. Another study from Johannesburg indicated that 10.4% of patients LTFU were hospitalized [28].

### Strengths and study implications

The Khayelitsha ART program is one of the largest and oldest public-sector ART programs in South Africa, lending credibility to our findings and conclusions. Additionally, we focused on a contemporary cohort, which allows us to draw conclusions that are applicable to current ART programs across the country, as well as in the region. The ability to track patients using a unique identifier across different health services and laboratory data, and link patients to the National Death Registry for mortality ascertainment [39], are distinctive within sub-Saharan Africa. This enabled us to provide robust estimates of true disengagement and reassurance that almost half of patients originally seen by the clinic as lost to follow-up are indeed either retained in care or returned to care. A study of the National Death Registry indicated that 94% of deaths were recorded by civil registration, lending support to the notion that those who we reported as truly disengaged from care and not registered as dead are most likely alive [8,39].

Potential interventions to improve patient tracking and retention, as well as provide a greater degree of flexibility in the system may include: rational use of patient held cards/records (which exist in Khayelitsha but are not always utilized), nationally accessible electronic health records, increasing the period for ART prescription to allow patient travel, editing clinical management protocols to include sections on transferring and receiving patients, improving the ability of clinics to communicate with each other and, perhaps most importantly, accepting that patients cycle through care and promoting healthcare worker understanding of the need to adapt care around patients’ lifestyles and mobility through health worker training [37].

### Limitations

We likely underestimate true silent transfer and return to care, as we only tracked patients in the Western Cape province. Because of the mobility of this population, it is likely that additional patients returned to care in other provinces, particularly the Eastern Cape. If person-level data were cascaded up to the national level in line with recommendations [14], and a unique health identifier was used nationally, it may be possible to track patient movements country-wide. Implementation of such a system has already been proposed by the South African National Department of Health [40]. We do recognize that patients who migrated from other countries may have poorer treatment outcomes due to mobility and legal status issues and would be less likely to have a national identification number and be in the death registry. Therefore, we may have underestimated mortality in this group.

We had a short period for follow-up. We were also unable to ascertain causes of death. As we included only patient follow-up from 2013–2014 by design in order to maximize the generalizability of our findings to current programs, this limited our ability to more fully describe temporal trends in first disengagement. Additionally, we recognize the nonuniform collection of data, which necessitated dropping particular variables from our analyses and performing multiple imputation for missing data. It is also possible that some clinic and/or lab visits were not captured in the database. This is somewhat mitigated by the large size of the dataset and power of the analysis.

Finally, the legacy of South African apartheid has contributed to specific mobility issues and social problems in communities such as Khayelitsha that may not be generalizable to other urban settings in sub-Saharan Africa.

### Conclusions

The Khayelitsha ART program is one of the oldest in South Africa and has grown greatly in size. The merging of all provincial and municipal clinic data in this study is unique and lends completeness to the cohort data as representative of an entire community. The ability to electronically trace patients throughout the Western Cape province and link to the National Death Registry allowed us to correct misclassified patient outcomes. For these reasons, the findings from this study are of value in directing appropriate patient and clinic-based interventions to improve long-term retention and meet the 90-90-90 HIV treatment targets in high prevalence urban settings such as this one throughout sub-Saharan Africa.

Our finding that many patients who disengaged return to care suggests that many patients in ART programs in Africa, particularly in urban settings, cycle in and out of care. This suggests a shift in the provision of ART care: that the linear UNAIDS model of HIV diagnosis, ART initiation, and viral suppression may need to be reconceptualized to account for this cycling in and out of care and the mobile populations served by ART programs in many sub-Saharan African countries. As “Universal Test and Treat” is implemented in both South Africa and the rest of sub-Saharan Africa, more patients will enroll in ART care with higher CD4 counts and need to be retained. It will be important to find ways to adapt services to accommodate mobile populations to retain patients in care and prevent morbidity, mortality, and HIV transmission. Even if these systems are slow to improve, clinics across sub-Saharan Africa that are familiar with and accommodating of the mobility of their patients will be able to better care and advocate for them.

## Supporting information

S1 STROBE Checklist Checklist for cohort study. (DOC)

S1 Protocol Original Substudy of protocol “Enhanced routine surveillance of patients in HIV care in Khayelitsha” (HREC 395/2005) entitled “A retrospective cohort and a nested case-control study of risk factors contributing to default (loss to follow up) from antiretroviral therapy (ART) care in Khayelitsha, South Africa”. (DOCX)

S1 Protocol Amendment Amendments to original protocol. (DOCX)

S1 Fig Examples of how each patient contributed time to survival analyses and Cox proportional hazards models. Bracketed dates indicate origin dates (first antiretroviral therapy [ART] visit); grey rectangles indicate entry date into the cohort.
(PNG)

S2 Fig Diagnostic plots for predictive mean matching used in imputation for variables with skewed distributions included in the Cox proportional hazards model. (PNG)

S3 Fig Proportional hazards plots for all variables included in the final imputed Cox proportional hazards model. (PDF)

S4 Fig **A. Kaplan-Meier estimate of time to return to care after disengagement, until 30 June 2015. B. Kaplan-Meier estimate of time to death after disengagement for those with national identification numbers who disengaged and were found to be dead in the National Death Registry, estimates until 30 June 2015**.
*For those who disengaged returning to care, the 180-day lag before patients return to care is a function of our definition that those who returned to care <180 days later were designated silent transfers and were censored on their date of return to care elsewhere in the Western Cape province.
*For death post-disengagement, the 3-month lag before patients die is a function of our definition to reclassify those who died ≤90 days after disengagement as deaths and were no longer classified as those who disengaged.
(TIF)

S1 Table South African ART eligibility criteria and management for adults and adolescents, over time [9,10,41–43]. AZT, zidovudine; ddI, didanosine; d4T, stavudine; DVR/r, dolutegravir/ritonavir; EPTB, extrapulmonary tuberculosis; EFV, efavirenz; FTC, emtricitabine; HBV, hepatitis B; m, months; LPV/r, lopinavir/ritonavir; MDR/XDR TB, multi-drug resistant / extensively drug resistant tuberculosis; NVP, nevirapine; RAL, raltegravir; TB, tuberculosis; TDF, tenofovir; 3TC, lamivudine; VL, viral load; WHO, World Health Organization.
(DOCX)

S2 Table Additional definitions. (DOCX)

S3 Table Patient characteristics by ART start date. * Variables/data available at provincial clinics only.
ABC, abacavir; AZT, zidovudine; d4T, stavudine; EFV, efavirenz; LPV/r, lopinavir/ritonavir; NVP, nevirapine; TB, tuberculosis; TDF, tenofovir.
(DOCX)

S4 Table Cox proportional hazard model sensitivity analyses. *Complete case analysis included missing data from the “adherence club” variable, as this variable was only included in the provincial databases. Excluding the missing data from this variable would have eliminated all municipal patients from the model. This model did not contain imputed data.
**This model contained imputed data but was restricted to those patients with national identification numbers to allow for accurate mortality ascertainment.
ABC, abacavir; ART, antiretroviral therapy; AZT, zidovudine; CI, confidence interval; d4T, stavudine EFV, efavirenz; LPV/r, lopinavir/ritonavir; NVP, nevirapine; ref, reference; TB, tuberculosis; TDF, tenofovir; yrs, years.
(DOCX)

S5 Table Overall final outcomes for silent transfers and those who disengaged, as locally ascertained by Western Cape province data systems and the National Death Registry. *Different from initial outcomes for those who disengaged as presented in Table 3.
(DOCX)

S6 Table Patient characteristics for those who disengaged and returned to care versus failed to return. * Variables/data available at provincial clinics only.
ABC, abacavir; AZT, zidovudine; d4T, stavudine; EFV, efavirenz; LPV/r, lopinavir/ritonavir; NVP, nevirapine; TB, tuberculosis; TDF, tenofovir.
(DOCX)

S7 Table Logistic regression model for odds of not returning to care, for those who disengaged (*n* = 7,167). *Those who returned to care included all patients who returned to a clinic in the Western Cape ≥180 days after their last visit. Those who did not return to care included patients who did not have any follow-up information found in the Western Cape databases. We excluded silent transfers, and patients who had disengaged but whose first data point after disengagement was either a) death or b) hospital contact.
**Variables selected for this model were the same variables selected for the multivariable Cox model in Table 2; however, CD4 variables were refined to more closely investigate a potential trend.
ART, antiretroviral therapy; CI, confidence interval; d4T, stavudine; EFV, efavirenz; LPV/r, lopinavir/ritonavir; NVP, nevirapine; ref, reference; TB, tuberculosis; yrs, years.
(DOCX)

## Abbreviations

ART: antiretroviral therapy
d4T: stavudine
GIS: geographic information system
HR: hazard ratio
LTFU: lost to follow-up
NHLS: National Health Laboratory Service
PEPFAR: the United States President’s Emergency Plan for AIDS Relief
PHA: proportional hazards assumption
WHO: World Health Organization
