# Supplementary material for: Contemporary disengagement from antiretroviral therapy in Khayelitsha, South Africa: A cohort study
Source: PLoS Med. 2017 Nov 7;14(11):e1002407. doi: 10.1371/journal.pmed.1002407 (PMC5675399; doi:10.1371/journal.pmed.1002407)
Supplement: S1 STROBE Checklist — (DOC) [file pmed.1002407.s001.doc]

STROBE Statement—Checklist of items that should be included in reports of ***cohort studies***

|  | Item No | Recommendation |
| --- | --- | --- |
| **Title and abstract** | 1 | (*a*) Indicate the study’s design with a commonly used term in the title or the abstract  Title: “…a cohort study” |
|  |  | (*b*) Provide in the abstract an informative and balanced summary of what was done and what was found  “Abstract” section |
| Introduction | | |
| Background/rationale | 2 | Explain the scientific background and rationale for the investigation being reported  “Introduction” section |
| Objectives | 3 | State specific objectives, including any prespecified hypotheses  Introduction, paragraph 3: “we sought to quantify disengagement from care and identify risk factors and outcomes for those patients who disengaged using electronic tracing methods.” |
| Methods | | |
| Study design | 4 | Present key elements of study design early in the paper  Data analysis, paragraph 1: “The cohort analysis was conducted in two parts: 1) analysis of incidence and time to disengagement from care in the cohort and analysis of risk factors for disengagement, using cumulative incidence curves and Cox proportional hazards models; and 2) for patients who disengaged from care, a description of outcomes after disengagement, and times to these outcomes.”  And, “Data analysis” section overall |
| Setting | 5 | Describe the setting, locations, and relevant dates, including periods of recruitment, exposure, follow-up, and data collection  Setting and inclusion criteria, paragraph 1: “A cohort study was conducted using data from all provincial and municipal public sector ART clinics (n=13) in Khayelitsha, Cape Town, South Africa.”  Data analysis, paragraph 2: “Entry into the study was at the beginning of 2013 for those who started ART prior to the analysis window, or at ART initiation if after 1 Jan 2013. Time to disengagement (failure) was defined as time from date of study entry to date of first disengagement within the window of 1 January 2013 – 31 December 2014 (Fig 2; S2 Table), Time origin was study entry, and in a second analysis, first ART start date, allowing for delayed entry into the survival analysis (Fig 3a). Date of disengagement was defined as the first date of the >=180 day period that the patient was not in care. While survival time (until disengagement) was only calculated through 31 December 2014, the database was closed at 30 June 2015 to allow disengagement ascertainment (i.e. the opportunity to meet the disengagement definition) for those whose last visit was in late 2014.”  Disengagement outcome analysis, paragraph 1: “For purposes of determining outcomes, the date of administrative censoring was extended to 30 June 2015.” |
| Participants | 6 | (*a*) Give the eligibility criteria, and the sources and methods of selection of participants. Describe methods of follow-up  Setting and inclusion criteria, paragraph 2: “The study included all patients who had at least one visit at a Khayelitsha ART clinic between 1 January 2013 – 31 December 2014 regardless of the date they initiated ART and provided it was prior to 31 December 2014. Patients not started on ART were excluded. This date range was selected to examine a contemporary cohort, but data included historical ART data prior to 2013 for patients selected for the cohort. Adults and adolescents age >=ten years of age by 1 January 2013 were included as we wanted to include adolescents in our analysis of risk factors for disengagement, and age ten is the start of adolescence as defined by the World Health Organization.” |
|  |  | (*b*) For matched studies, give matching criteria and number of exposed and unexposed  N/A |
| Variables | 7 | Clearly define all outcomes, exposures, predictors, potential confounders, and effect modifiers. Give diagnostic criteria, if applicable  “Key definitions” section, S2 Table  Cumulative incidence estimates and Cox proportional hazards model for disengagement, paragraph 1: “Possible outcomes for patients as of 31 December 2014 were 1) alive and in care; 2) dead; 3) transferred out; or 4) disengaged. The primary outcome was time to disengagement during the two-year window of analysis. Secondary analyses focused on risk factors associated with disengagement.”  Disengagement outcome analysis, paragraph 1: “Possible primary outcomes for those who disengaged were: 1) death; 2) return to care at a different facility within 180 days (silent transfer); 3) return to care after 180 days; 4) hospital admission; 5) other hospital contact (outpatient or emergency visit); 6) alive on 30 June 2015 if they had a national identification number but no death date was found in the National Death Registry; or 7) no information; still disengaged. Primary outcomes were identified as the **first** of these outcomes after the date of disengagement. Secondary outcomes were time to return to care and time to death after disengagement.” |
| Data sources/ measurement | 8* | For each variable of interest, give sources of data and details of methods of assessment (measurement). Describe comparability of assessment methods if there is more than one group  “ART eligibility, monitoring, and treatment regimens” section; “Data collection and management” section |
| Bias | 9 | Describe any efforts to address potential sources of bias  “Limitations” sub-section |
| Study size | 10 | Explain how the study size was arrived at  Setting and inclusion criteria, paragraph 2: “The study included all patients who had at least one visit at a Khayelitsha ART clinic between 1 January 2013 – 31 December 2014 regardless of the date they initiated ART provided it was prior to 31 December 2014. Patients not started on ART were excluded. This date range was selected to examine a contemporary cohort, but data included historical ART data prior to 2013 for patients selected for the cohort. Adults and adolescents age >=ten years of age by 1 January 2013 were included as we wanted to include adolescents in our analysis of risk factors for disengagement, and age ten is the start of adolescence as defined by the World Health Organization.” |
| Quantitative variables | 11 | Explain how quantitative variables were handled in the analyses. If applicable, describe which groupings were chosen and why  Table 2. |
| Statistical methods | 12 | (*a*) Describe all statistical methods, including those used to control for confounding  “Data analysis” section |
|  |  | (*b*) Describe any methods used to examine subgroups and interactions  Cumulative incidence estimates and Cox proportional hazards model for disengagement, paragraph 6: “In secondary analyses, we report results for a complete case analysis excluding missing data, as well as an imputed model restricted to patients with national identification numbers, which permitted linking to the National Death Registry.”  Disengagement outcome analysis, paragraph 1: “We conducted a sensitivity analysis of disengagement outcomes for those with national identification numbers to restrict analyses to only those with reliable vital status ascertainment.” |
|  |  | (*c*) Explain how missing data were addressed  Cumulative incidence estimates and Cox proportional hazards model for disengagement, paragraph 6: “We used imputation by chained equations to impute missing data for TB, weight, club participation, CD4 count, HIV viral load, and drug regimens five times. The imputation model included all measured variables, used predictive mean matching for variables with skewed distributions (CD4 count, log HIV viral load), and used multinomial logistic regression for binary and categorical variables.” |
|  |  | (*d*) If applicable, explain how loss to follow-up was addressed  N/A; subject of manuscript |
|  |  | (*e*) Describe any sensitivity analyses  Cumulative incidence estimates and Cox proportional hazards model for disengagement, paragraph 6: “The primary modeling results are based on the imputed data. In secondary analyses, we report results for a complete case analysis excluding missing data, as well as an imputed model restricted to patients with national identification numbers, which permitted linking to the National Death Registry.”  Disengagement outcome analysis, paragraph 1: “We conducted a sensitivity analysis of disengagement outcomes for those with national identification numbers to restrict analyses to only those with reliable vital status ascertainment.” |
| Results | | |
| Participants | 13* | (a) Report numbers of individuals at each stage of study—eg numbers potentially eligible, examined for eligibility, confirmed eligible, included in the study, completing follow-up, and analysed  Figure 1, results paragraph 1: “A total of 53,461 patients initiated ART at any Khayelitsha site since program inception through 31 December 2014. For the cohort study, we excluded 11,839 patients who did not have a visit in the time period between 1 January 2013 and 31 December 2014, and 1,607 who were less than ten years old at 1 January 2013. An additional 131 were excluded due to incomplete data at the primary data collection level (Fig 1).” |
|  |  | (b) Give reasons for non-participation at each stage  Figure 1 |
|  |  | (c) Consider use of a flow diagram  Figure 1 |
| Descriptive data | 14* | (a) Give characteristics of study participants (eg demographic, clinical, social) and information on exposures and potential confounders  Table 1, S3 Table |
|  |  | (b) Indicate number of participants with missing data for each variable of interest  Table 1, S3 Table |
|  |  | (c) Summarise follow-up time (eg, average and total amount)  Results, paragraph 1: “Of the 39,884 patients remaining (Table 1), the median follow-up from ART start date to 31 December 2014 was 33.6 months (IQR 12.4-63.2).” |
| Outcome data | 15* | Report numbers of outcome events or summary measures over time  Fig 2, Fig 3a, Fig 3b, Fig 3c, Table 3, S4a Fig, S4b Fig, S5 Table  Results, paragraph 3: “As of 31 December 2014, of the total cohort, 592 (1.5%) died, 1,231 (3.1%) transferred out, 987 (2.5%) were silent transfers and visited another ART or primary care clinic in the same province (Western Cape) within 180 days of their last visit date, 9,005 (22.6%) disengaged, and 28,069 (70.4%) were in care…Total mortality for the entire cohort as of 30 June 2015 was 2.4% (n=939), and 3.9% (n=822) when restricted to those with national identification numbers…. The cumulative incidence of disengagement from care, before any other event could occur, was 25.1% at two years, analyzed by time in the study (Fig 2). The cumulative incidence of disengagement was 50.3% and 60.3% at five and ten years on ART respectively (Fig 3a), based on the person time contributed during the analysis window but analyzed relative to ART initiation date. The higher hazard of disengagement soon after starting ART was largely attenuated after adjusting for the patient characteristics included in Table 2, without evidence of a temporal effect comparing 2013 to 2014 (Fig 3b and 3c).” |
| Main results | 16 | (*a*) Give unadjusted estimates and, if applicable, confounder-adjusted estimates and their precision (eg, 95% confidence interval). Make clear which confounders were adjusted for and why they were included  Fig 2, Fig 3a, Table 2, S7 Table  Factors associated with disengagement, paragraph 1: “The strongest adjusted associations with disengagement were most recent CD4 count <350 cells/μl (CD4 200-350 hazard ratio (HR) 2.03; 95% CI 1.91-2.15; CD4 50-200 HR 3.07; 95% CI 2.84-3.31; CD4 <50 HR 3.34; 95% CI 2.92-3.83, all relative to CD4 > 350), use of d4T (stavudine) at last visit (HR 1.72; 95% CI 1.57-1.89), and pregnancy at ART start (HR 1.58; 95% CI 1.47-1.69) (Table 2).” |
|  |  | (*b*) Report category boundaries when continuous variables were categorized  Table 2, S7 Table |
|  |  | (*c*) If relevant, consider translating estimates of relative risk into absolute risk for a meaningful time period  N/A |
| Other analyses | 17 | Report other analyses done—eg analyses of subgroups and interactions, and sensitivity analyses  S2 Fig, S3 Fig, S4 Table, Table 3, S6 Table, S7 Table  Factors associated with disengagement, paragraph 2: “Post-imputation distributions for variables with skewed distributions were compared with observed distributions and were acceptable (S2 Fig). Proportional hazard assumptions for imputed variables were also met, with only a small deviation for long follow-up for ART regimen drug 3 (S3 Fig). Sensitivity analyses restricted to complete data or to patients with national identification numbers did not materially alter these associations (S4 Table).”  Outcomes after disengagement, paragraph 1: “Because only 60.7% (n=5,463) of those who disengaged had valid national identification numbers that could be linked to the National Death Registry, we conducted a sensitivity analysis of only these patients (Table 3).” |
| Discussion | | |
| Key results | 18 | Summarise key results with reference to study objectives  Discussion, paragraph 1: “In this study, we examined disengagement from ART care in 2013-2014 among patients of the large, peri-urban cohort in Khayelitsha: one of the oldest public sector ART cohorts in South Africa. Roughly one in five patients disengaged from care, demonstrating a high rate of disengagement and a key challenge to reaching the UNAIDS 90-90-90 treatment targets. Factors associated with disengagement were age <30 years, male sex, pregnancy at ART initiation, and last CD4 count <350 cells/μl. Factors associated with retention were ART adherence club membership and baseline CD4 <350 cells/μl. However, despite the high incidence of disengagement, many of those who disengaged did not do so permanently. While 48% of patients could not be traced (either did not have a national identification number or had an ID number and/or medical record number but no additional data were found), and 16% were admitted to the hospital at some point after disengagement, roughly one in three patients returned to care within the province during the study period, and half were estimated to return to care within 2.5 years. Additionally, not included in the overall estimate of disengagement are the 2.5% of silent transfers who appeared to disengage from a clinic perspective, but were actually in care elsewhere in the Western Cape province when province-wide data linkage was performed. These data indicate that a high proportion of patients are cycling in and out of care as well as transferring elsewhere in the province (often “silently”), and potentially to facilities outside of the Western Cape province (something that our study could not ascertain).” |
| Limitations | 19 | Discuss limitations of the study, taking into account sources of potential bias or imprecision. Discuss both direction and magnitude of any potential bias  “Limitations” section |
| Interpretation | 20 | Give a cautious overall interpretation of results considering objectives, limitations, multiplicity of analyses, results from similar studies, and other relevant evidence  “Conclusions” section |
| Generalisability | 21 | Discuss the generalisability (external validity) of the study results  “Conclusions” section |
| Other information | | |
| Funding | 22 | Give the source of funding and the role of the funders for the present study and, if applicable, for the original study on which the present article is based  Included in online PLOS Medicine submission.  “SK was supported by the National Institutes of Health Office of the Director, Fogarty International Center, Office of AIDS Research, National Cancer Center, National Heart, Blood, and Lung Institute, and the NIH Office of Research for Women’s Health through the Fogarty Global Health Fellows Program Consortium comprised of the University of North Carolina, John Hopkins, Morehouse and Tulane (R25TW009340).  GM was supported by the Wellcome Trust (098316), the EDCTP (SP.2011.41304.074) the South African Research Chairs Initiative of the Department of Science and Technology and National Research Foundation (NRF) of South Africa (Grant No 64787), NRF incentive funding (UID: 85858) and the South African Medical Research Council through its TB and HIV Collaborating Centres Programme with funds received from the National Department of Health (RFA# SAMRC-RFA-CC: TB/HIV/AIDS-01-2014).  AB was supported by the NIH (R01 HD080465, U01 AI069924) and NRF incentive funding.  The funders had no role in study design, data collection and analysis, decision to publish, or preparation of the manuscript.” |

*Give information separately for exposed and unexposed groups.

**Note:** An Explanation and Elaboration article discusses each checklist item and gives methodological background and published examples of transparent reporting. The STROBE checklist is best used in conjunction with this article (freely available on the Web sites of PLoS Medicine at http://www.plosmedicine.org/, Annals of Internal Medicine at http://www.annals.org/, and Epidemiology at http://www.epidem.com/). Information on the STROBE Initiative is available at http://www.strobe-statement.org.
