# Supplementary material for: Contemporary disengagement from antiretroviral therapy in Khayelitsha, South Africa: A cohort study
Source: PLoS Med. 2017 Nov 7;14(11):e1002407. doi: 10.1371/journal.pmed.1002407 (PMC5675399; doi:10.1371/journal.pmed.1002407)
Supplement: S1 Protocol Original — (DOCX) [file pmed.1002407.s002.docx]

**APPROVED 6 AUGUST 2015**

***Sub-study of protocol “Enhanced routine surveillance of patients in HIV care in Khayelitsha” (HREC 395/2005)***

**Title**: A retrospective cohort and a nested case-control study of risk factors contributing to default (loss to follow up) from ART care in Khayelitsha, South Africa

**Background:**

As of December 2013, over 9 million people in Sub-Saharan Africa were receiving antiretroviral therapy (ART), representing a dramatic success for the ART roll-out that began in the early 2000s. However, this represents only 37% of all people living with HIV in Sub-Saharan Africa.^1^ There remains an urgent need to expand testing and treatment services to all patients requiring treatment and to retain those in care who are receiving ART. In Khayelitsha township outside of Cape Town, South Africa, an HIV treatment program was established in 2000 at three public sector primary care clinics serving 500,000 people. The program has since expanded to 11 clinics supplying ART to >30,000 patients. While the cumulative mortality of patients at one year of ART has decreased from 14.8% in 2001 to 1.7% in 2009, challenges remain in long -term provision of ART.^2^ As of 2009 in Khayelitsha, 87% of ART patients were alive and on ART at 12 months, 75.2% at 36 months, and 65% at 6 years, highlighting that strategies to retain patients in care could still improve.^2^ Analyses of the program from 2001-2007 indicated that 33% of persons lost to follow-up (LTFU) had in fact died. After correction for mortality, rates of LTFU at one year (in persons who had not died) increased from 0% in 2001 and 2003 to 1.7% in 2004, 3.8% in 2005, and 7.6% in 2007.^3,4^ Further data from 2010-2012 indicate that the probability of being LTFU increased during this period compared to previous calendar periods.^5^ In order to address this issue of LTFU in Khayelitsha, it is necessary to identify modifiable risk factors for default from ART and the outcomes of these patients so that interventions can be appropriately targeted to improve long–term retention and reduce mortality in this patient population.

The current UCT HRC approved protocol “*Enhanced routine surveillance of patients in HIV care in Khayelitsha*” outlines the unique contribution of the Khayelitsha cohort data in light of Khayelitsha’s status as one of the longest standing and largest HIV treatment programmes in South Africa. The proposed sub-study falls under the purview of continued collaborative analyses to assist in service delivery in Khayelitsha and other areas around South Africa. Linkage of the Khayelitsha cohort to mortality data (as approved by HREC 137/2008) will also contribute to this aim.

**Aims and Objectives / Purpose of the Study**

In a meta-analysis of 15 ART programs in sub-Saharan Africa, Brinkhof et al. (2009) found that the percentage of patients lost to the programs ranged from 5 to 50% and mortality ranged from 12-87% of patients LTFU. In addition to deaths, the most common reasons for patients not returning to clinic were financial problems, deteriorating or improving health, and transfer to another program. In a qualitative study from South Africa, Miller et al (2010) found that transport and logistical barriers in clinics (including patient-friendly clinic hours) contributed to default from ART care. In line with this previous research, our hypothesis for this study is that logistical barriers in clinics, as well as individual demographic and health factors contribute to loss to follow up from ART care and subsequent morbidity and mortality, as measured by hospitalizations and vital status.

The specific aims of this study will be to 1) identify the clinics at which the highest incidence of default is occurring in Khayelitsha, 2) identify risk factors for default from ART care, and 3) determine the outcomes of patients who default on ART. In doing so, this study will aim to identify both patient and clinic factors for which future interventions could be directed to reduce default (see below for a list of potential risk factors).

**Methodology**

*Study Design:*

Default from ART care will be defined as failing to attend a follow-up appointment at an ART clinic > three months after the last appointment. We will perform two inter-related studies:

1) Retrospective cohort study**:** The cohort will be all patients attending ART clinics in Khayelitsha at least once between 1 January 2014 – 31 December 2014. This cohort will include persons who have been on ART for varying lengths of time. We will determine the incidence of default from ART care during this time period in Khayelitsha as a whole and by ART clinic, allowing a 3- month buffer into 2015 to those patients presenting to clinics late in the calendar year to allow for the possibility of default during this time. We will describe the demographic and clinical characteristics of the patients who default and the distribution of durations on ART when default occurs. Utilizing clinic records and databases, the national death register, and the ongoing Khayelitsha cohort study directed by one of my mentors Andrew Boulle, we will also determine outcomes of the patients who defaulted in terms of return to another ART clinic in Khayelitsha, admission to the Khayelitsha Hospital (and admission diagnosis), death, and the timing of these outcomes in relation to ART default. The use of a unique identifier across the different electronic data platforms that are used to capture clinical data from the hospital and HIV and TB clinics in Khayelitsha will allow us to link databases and establish these outcomes.

2) Nested case-control study: To determine risk factors for LTFU, we will randomly select 200 patients who defaulted ART, and select one control patient (who did not default ART) for each case, matched for ART clinic and calendar year in which the patient started ART. We will extract from the clinic records for these cases and controls demographic and clinic variables (e.g., age, gender, area of residence, marital status, employment status, baseline CD4, ART prescribed, first versus second line ART, ART side effects, TB treatment, psychological complaints, substance abuse, physical symptoms, collecting medication monthly or every 3 months, belonging to a treatment support club or not). Other variables such as economic factors are not currently in the databases and will not be able to be included in the analysis.

*Sample size assumptions and estimates:*  Nested case-control study: In order to detect an odds ratio of 2.5 for variables that have a prevalence of 10% among controls (e.g. second line ART or alcohol abuse, both of which have prevalence of ~10% in this cohort based on previous studies), a sample size of 388 (194 pairs) is required (alpha = 0.05; power = 0.90). We therefore plan to include 200 pairs in this study. There are over 30,000 adult patients on ART in Khayelitsha. Given the percentage of patients defaulting ART described above, we anticipate sufficient numbers for this study.

*Characteristics of the study population:* The study population will comprise all patients on ART in the Khayelitsha clinic system of over 30,000 patients. The research will be retrospective, based at the University of Cape Town, with some travel to the Khayelitsha clinics to obtain missing data.

*Recruitment and enrollment of participants:* We will utilize patient records from electronic and paper data management systems that cover the clinics in Khayelitsha.

*Research procedures and data collection methods:* Data will be extracted from existing electronic and paper medical and hospital records, and the national death registry. Patients will not be contacted nor will informed consent be obtained due to the anonymity of the study.

*Intervention:* This study aims to identify risk factors at which future interventions can be directed in the Khayelitsha clinics to reduce default from ART care. This specific study will not seek to carry out any interventions, but the manuscripts emanating from the study will propose future interventions. Andrew Boulle holds a position in the provincial government and this will assist in reporting findings back to policy makers.

*Data analysis plan:* The data will be analyzed using STATA and GraphPad Prism software programs. For the case-control study, for each exposure variable we will calculate the odds ratio for default in univariate analysis. Variables associated with the outcome with p-value <0.1 and certain *a priori* variables will be entered into multivariate logistic regression models to determine independent risk factors for ART default.

**Informed consent process**

Informed consent will not be obtained for this study, as it is retrospective and anonymous in nature.

**Privacy and Confidentiality**

Please see section in original protocol outlining the protection of the electronic patient information systems in use for this study.

**Resources**

This study will be funded by the National Institutes of Health, Fogarty Center, through a pre-doctoral grant awarded to Samantha Kaplan, co-investigator.

**References**

1. World Health Organization. Global update on the health sector response to HIV, 2014. Accessed 24 Nov 2014 at: http://www.who.int/hiv/pub/progressreports/update2014/en/
2. Medecins Sans Frontieres. Khayelitsha Activity Report 2001-2011: 10 Years of HIV/TB Care at Primary Health Care Level. Accessed 23 Nov 2014 at: http://www.msf.org/article/khayelitsha-activity-report-2001-2011-10-years-hivtb-care-primary-health-care-level
3. Boulle A et al. Seven-year experience of a primary care antiretroviral treatment programme in Khayelitsha, South Africa. AIDS. 2010; 24:563-572.
4. Van Cutsem et al. Correcting for Mortality Among Patients Lost to Follow Up on Antiretroviral Therapy in South Africa: A Cohort Analysis. PLoS One. 2011; 6(2):e14684.
5. Stinson et al. 12-year experience of a primary care antiretroviral treatment programme in Khayelitsha, South Africa. Poster presented at 2013 ICASA Conference, Cape Town, S. Africa, 7-11 Dec 2013.
6. Brinkhof MW et al. Mortality of patients lost to follow-up in antiretroviral treatment programmes in resource-limited settings: Systematic review and meta-analysis. PLoS One. 2009 Jun 4;4(6):e5790.
7. Miller CM et al. Why are antiretroviral treatment patients lost to follow-up? A qualitative study from South Africa. Trop Med Int Health. 2010 Jun;15 Suppl 1:48-54.
