# Supplementary material for: Contemporary disengagement from antiretroviral therapy in Khayelitsha, South Africa: A cohort study
Source: PLoS Med. 2017 Nov 7;14(11):e1002407. doi: 10.1371/journal.pmed.1002407 (PMC5675399; doi:10.1371/journal.pmed.1002407)
Supplement: S1 Protocol Amendment — (DOCX) [file pmed.1002407.s003.docx]

**Amendments made to S1 protocol**

******All amendments/changes were made organically throughout the dataset creation and data analysis processes; there was no specific date for each decision. We created our dataset from existing retrospective data and changed parts of the analysis as this dataset was created and we were able to more accurately calculate the number of patients who would be included in each analysis and sub-analysis.*

**1.** Dates of ART clinic attendance were changed to 1 January 2013 – 31 Dec 2014 (from 1 January 2014 to 31 Dec 2014 as stated in the protocol) to increase the sample size of patients included in the study.

2. “Disengagement” definition was changed from 3 months (as stated in the protocol) to 6 months without a clinic visit to more closely align with previous studies as outlined in the methods section of the manuscript.

3. To examine risk factors for disengagement, we used a multivariable Cox proportional hazards model instead of a nested case-control study as stated in the protocol, as we determined that we would not be able to reliably abstract additional data on potential associations and confounders which were not already routinely available.

4. We used multiple imputation to impute missing data in order to increase our sample size and power of analysis for the Cox proportional hazards model.

5. We added a GIS analysis of locations where patients who disengaged returned to care in order to visually illustrate how patients may move across the Western Cape province and re-engage in care.

6. In response to reviewer comments, we conducted an additional analysis using a logistic regression model to determine associations with failure to re-engage in care after disengagement.
