# Supplementary figures and images for: Contemporary disengagement from antiretroviral therapy in Khayelitsha, South Africa: A cohort study
Source: PLoS Med. 2017 Nov 7;14(11):e1002407. doi: 10.1371/journal.pmed.1002407 (PMC5675399; doi:10.1371/journal.pmed.1002407)

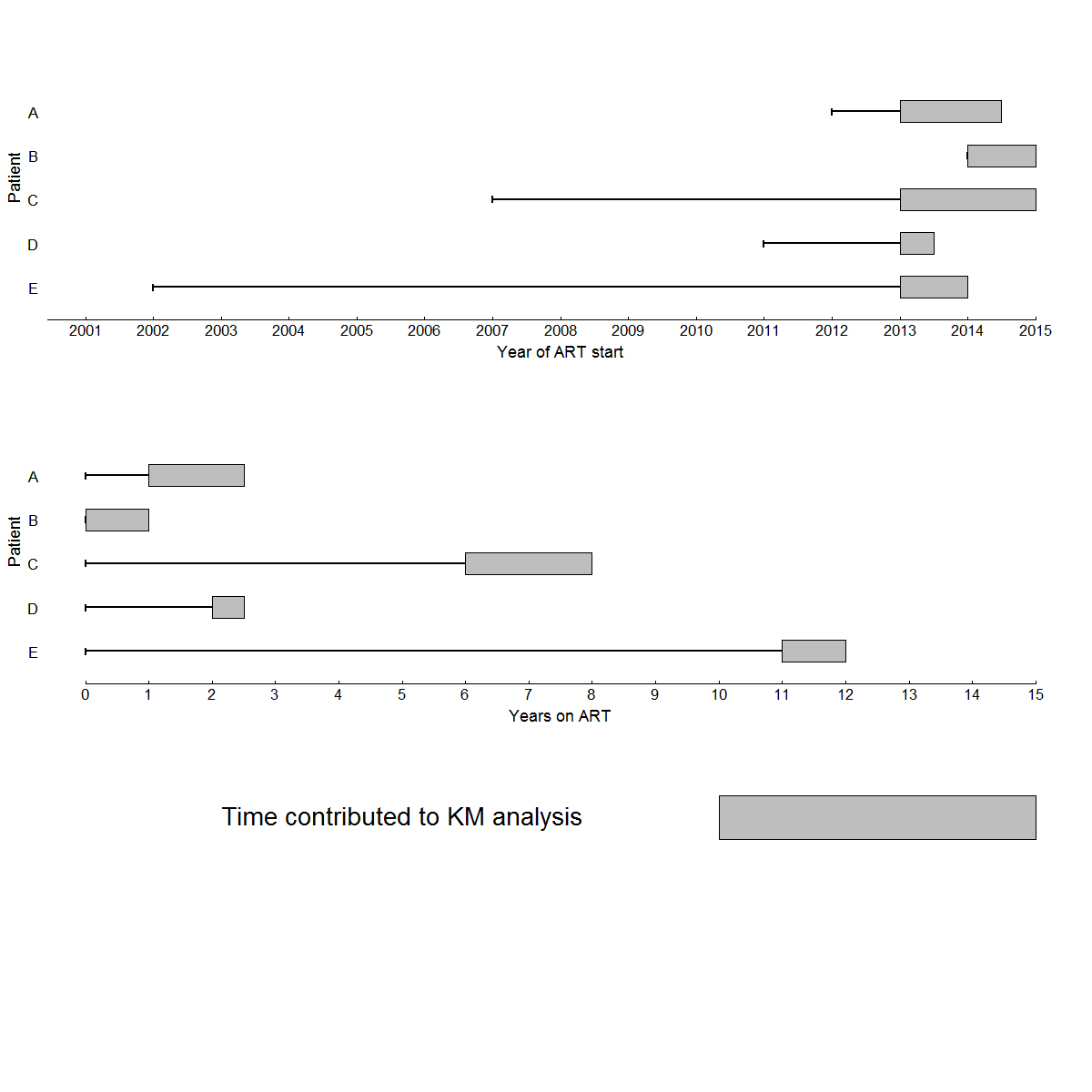

Supplement: S1 Fig — Bracketed dates indicate origin dates (first antiretroviral therapy [ART] visit); grey rectangles indicate entry date into the cohort. (PNG) [file pmed.1002407.s004.png]

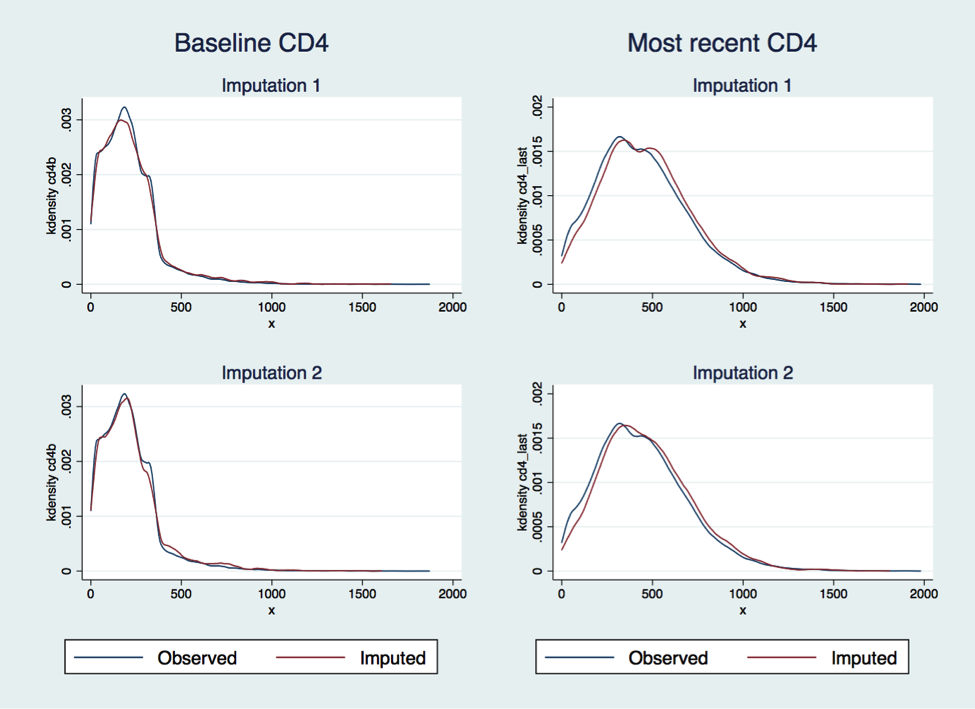

Supplement: S2 Fig — (PNG) [file pmed.1002407.s005.png]

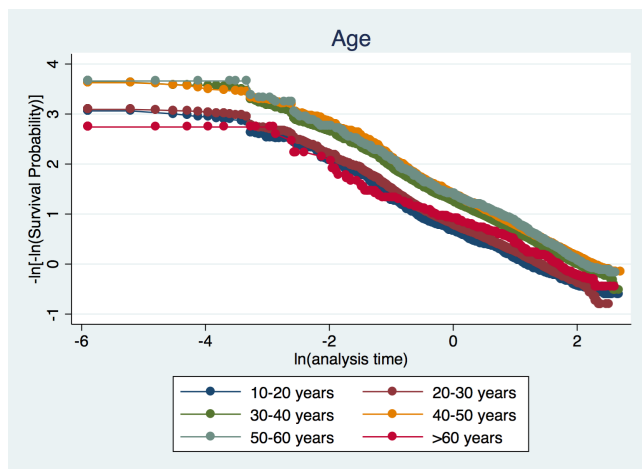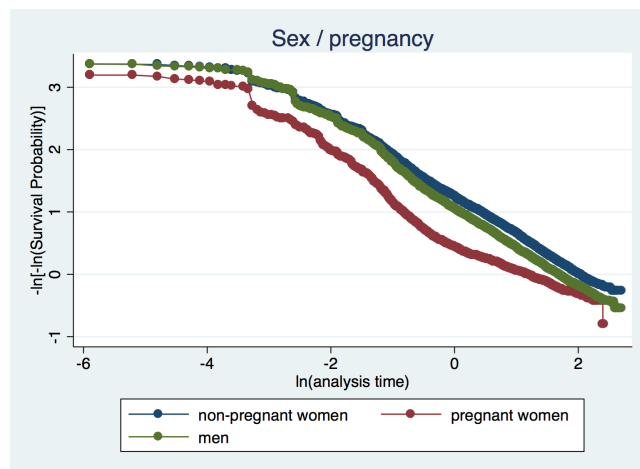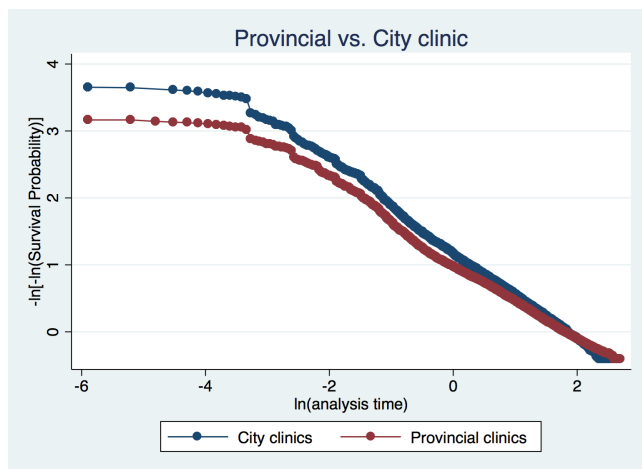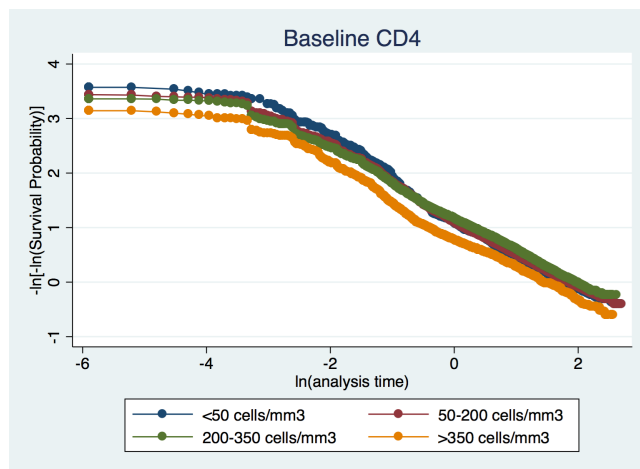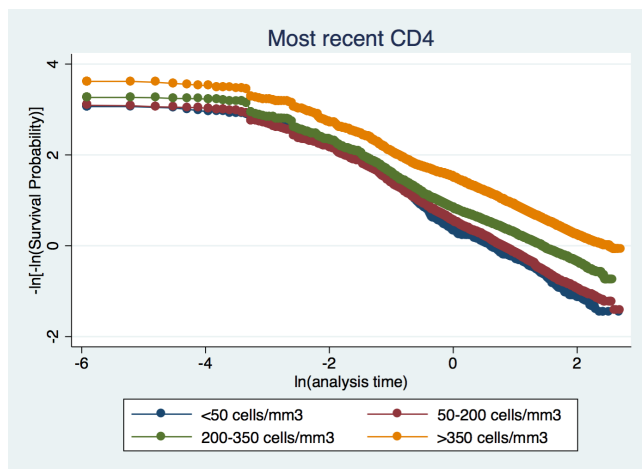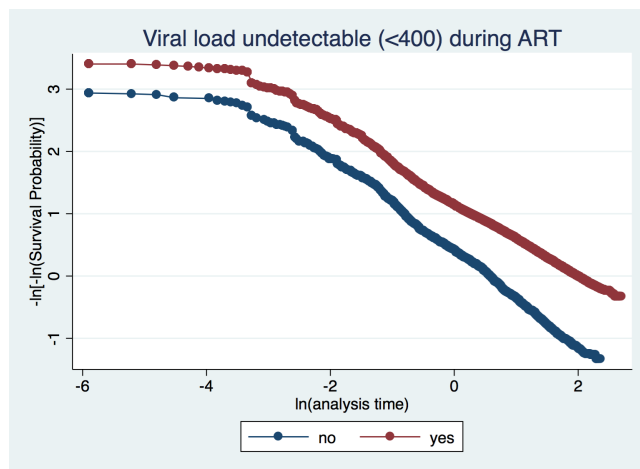

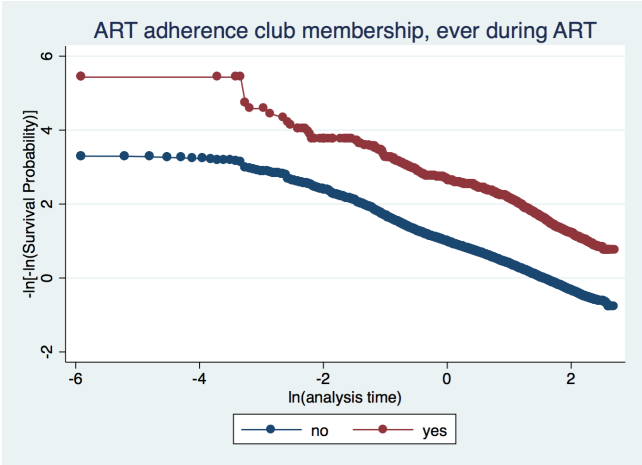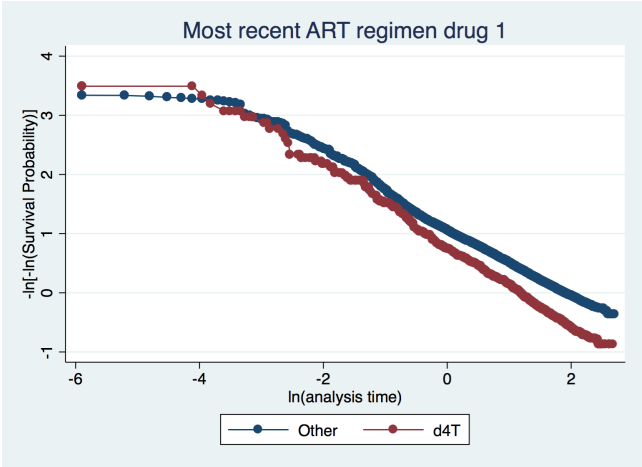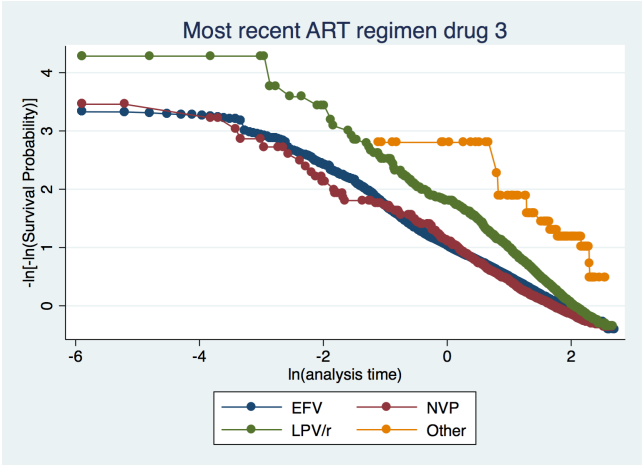

Supplement: S3 Fig — (PDF) [file pmed.1002407.s006.pdf]

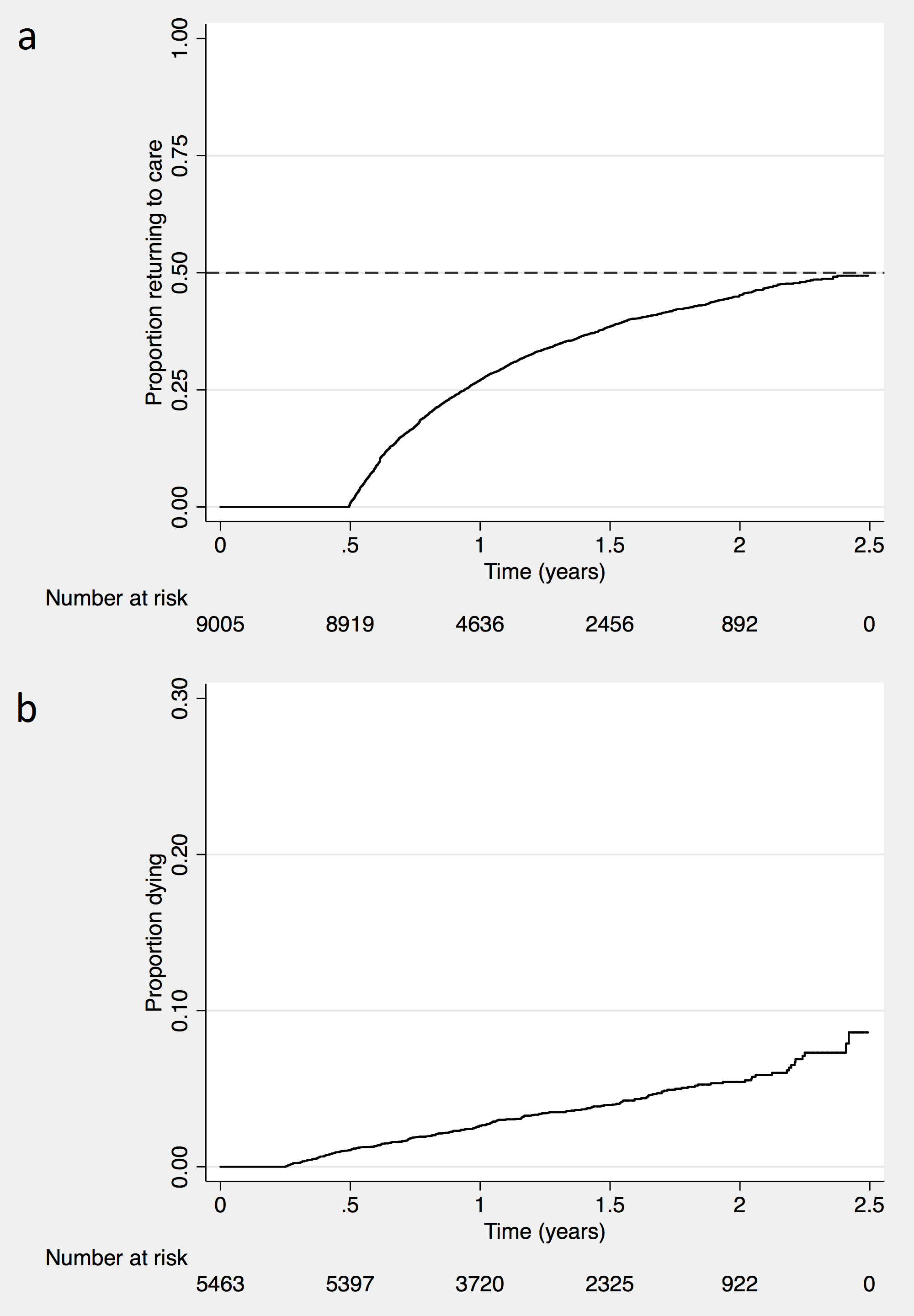

Supplement: S4 Fig — A. Kaplan-Meier estimate of time to return to care after disengagement, until 30 June 2015. B. Kaplan-Meier estimate of time to death after disengagement for those with national identification numbers who disengaged and were found to be dead in the National Death Registry, estimates until 30 June 2015. *For those who disengaged returning to care, the 180-day lag before patients return to care is a function of our definition that those who returned to care <180 days later were designated silent transfers and were censored on their date of return to care elsewhere in the Western Cape province. *For death post-disengagement, the 3-month lag before patients die is a function of our definition to reclassify those who died ≤90 days after disengagement as deaths and were no longer classified as those who disengaged. (TIF) [file pmed.1002407.s007.tif]
