# Supplementary material for: Contemporary disengagement from antiretroviral therapy in Khayelitsha, South Africa: A cohort study
Source: PLoS Med. 2017 Nov 7;14(11):e1002407. doi: 10.1371/journal.pmed.1002407 (PMC5675399; doi:10.1371/journal.pmed.1002407)
Supplement: S1 Table — AZT, zidovudine; ddI, didanosine; d4T, stavudine; DVR/r, dolutegravir/ritonavir; EPTB, extrapulmonary tuberculosis; EFV, efavirenz; FTC, emtricitabine; HBV, hepatitis B; m, months; LPV/r, lopinavir/ritonavir; MDR/XDR TB, multi-drug resistant / extensively drug resistant tuberculosis; NVP, nevirapine; RAL, raltegravir; TB, tuberculosis; TDF, tenofovir; 3TC, lamivudine; VL, viral load; WHO, World Health Organization. (DOCX) [file pmed.1002407.s008.docx]

**S1 Table. South African ART eligibility criteria and management for adults and adolescents, over time (9,** [**10**](#_ENREF_10)**, 41-43)**

|  |  | **May 2001** | **April 2004** | **April 2010** | **April 2013** | **April 2015** |
| --- | --- | --- | --- | --- | --- | --- |
| **Eligibility** | ***Stage*** | WHO IV (excluding EPTB) | WHO IV (excluding EPTB) | WHO IV | WHO III or IV | WHO III or IV |
|  | ***CD4 count*** | 200 cells/ μl | 200 cells/ μl | 350/ μl for TB/pregnancy; 200 for other adults | 350/ μl in other adults | 500 cells/ μl |
|  | ***Other*** |  |  | MDR/XDR TB | Sero-discordant couples; all with TB/pregnancy or breastfeeding | All with TB/pregnancy or breastfeeding or known HBV |
| **Regimen** | ***First-line*** | AZT, 3TC, NVP \| EFV | D4T,3TC, NVP\|EFV | TDF,FTC\|3TC,EFV\|NVP | FDC (TDF,FTC,EFV) preferred | Unchanged |
|  | ***Second-line*** | D4T,ddI,NFV\|IDV\|SQV | AZT,ddI,LPV/r | TDF\|AZT,3TC\|FTC,LPV/r | Unchanged | Unchanged |
|  | ***Third-line*** |  |  |  | RAL,DVR/r,ETR |  |
| **Monitoring** | ***Viral load*** | Baseline, 3, 6, 12m and annual | Baseline, 6, 12 m, and annual | 6m, 12m and annual | 4m, 12m, and annual | 6m, 12m, annually |
|  | ***CD4 count*** | Baseline, 6, 12m and annual | Baseline, 6, 12 m and annual | Unchanged | Baseline, 6m, 12m | Baseline, 12m, then annually if indicated |
|  | ***Failure definition*** | 2 x vl > 5000/ml | 2 x VL >=400/ml, 2^nd^ >=5000/ml | 2 x VL>=1000 | Unchanged | Unchanged |

*AZT, zidovudine; ddI, didanosine; d4T, stavudine; DVR/r, dolutegravir/ritonavir; EFV, efavirenz; EPTB, extrapulmonary tuberculosis; ETR, etravirine; FTC, emtricitabine; HBV, hepatitis B; IDV, indinavir; LPV/r, lopinavir/ritonavir; MDR/XDR TB, multi-drug resistant / extensively drug resistant tuberculosis; m, months; NFV, nelfinavir; NVP, nevirapine; RAL, raltegravir; SQV, saquinavir; TB, tuberculosis; TDF, tenofovir; 3TC, lamivudine; VL, viral load; WHO, World Health Organization*
