# Supplementary material for: Contemporary disengagement from antiretroviral therapy in Khayelitsha, South Africa: A cohort study
Source: PLoS Med. 2017 Nov 7;14(11):e1002407. doi: 10.1371/journal.pmed.1002407 (PMC5675399; doi:10.1371/journal.pmed.1002407)
Supplement: S2 Table — (DOCX) [file pmed.1002407.s009.docx]

**S2 Table. Additional definitions**

| ART adherence club | Entry into club requires patients to be stable on ART, meaning that they have attended regular clinic visits for >12 months and are virally suppressed. Clubs are managed by lay health workers and meet five times a year, allowing ease of medication refills by lay health workers without long lines for doctor visits, as well as offering communal peer support |
| --- | --- |
| Baseline CD4 count | Any CD4 count result available from 183 days prior to 7 days post ART initiation. If more than one measure was available, the CD4 count prior to and closest to ART initiation was used |
| “Most recent” CD4 or HIV viral load | The result at the most recent date within 13 months prior to disengagement or end of the study period (31 December 2014). No lab values from 2015 were included |
| Previously disengaged | Patient who had a gap in care of >180 days prior to 1 Jan 2013; included as a covariate |
| Return to care | Any evidence of a CD4 count, viral load, ART prescription, or primary care or ART clinic visit after disengagement, either in Khayelitsha or elsewhere in the Western Cape Province |
| “Suppressed” viral load | <400 copies/ml |
| Transfer out | Transfer to another clinic outside of Khayelitsha, as identified and documented by Khayelitsha clinic staff |
