# Supplementary material for: Contemporary disengagement from antiretroviral therapy in Khayelitsha, South Africa: A cohort study
Source: PLoS Med. 2017 Nov 7;14(11):e1002407. doi: 10.1371/journal.pmed.1002407 (PMC5675399; doi:10.1371/journal.pmed.1002407)
Supplement: S3 Table — * Variables/data available at provincial clinics only. ABC, abacavir; AZT, zidovudine; d4T, stavudine; EFV, efavirenz; LPV/r, lopinavir/ritonavir; NVP, nevirapine; TB, tuberculosis; TDF, tenofovir. (DOCX) [file pmed.1002407.s010.docx]

**S3 Table. Patient characteristics by ART start date**

|  | **Those who started ART prior to 1 January 2013** | | | **Those who started ART after 1 January 2013** | | |
| --- | --- | --- | --- | --- | --- | --- |
| **# of participants (*n*)** | 25,864 | # of patients with complete data | % complete data | 14,020 | # of patients with complete data | % complete data |
| **Age at 1 Jan 2013, years (median, IQR)** | 36.2 (30.4-42.5) | 25,864 | 100.0% | 30.8 (25.7-37.6) | 14,020 | 100.0% |
| **Male sex (*n*, %)** | 7,713 (29.8%) | 25,864 | 100.0% | 3,974 (28.4%) | 14,020 | 100.0% |
| **Months on ART at 1 Jan 2013 (median, IQR)** | 31 (13.5-55.7) | 25,864 | 100.0% | - | - | - |
| **Months on ART at 31 Dec 2014 (median, IQR)** | 52.6 (34.7-78.0) | 25,864 | 100.0% | 7.8 (3.2-14.9) | 14,020 | 100.0% |
| **Median year of ART start (median, IQR)** | 2010 (2008-2011) | 25,864 | 100.0% | - | - | - |
| **Baseline CD4 count, cells/μl (median, IQR)** | 165 (89-235) | 22,656 | 87.6% | 255 (146-344) | 12,557 | 89.6% |
| **CD4 count at 1 Jan 2013, cells/μl (median, IQR)** | 378 (248-544) | 25,191 | 97.4% | - | - | - |
| **Most recent CD4 count as of 31 Dec 2014, cells/μl (median, IQR)** | 480 (323-646) | 21,842 | 84.4% | 309 (192-464) | 12,653 | 90.2% |
| **Maximum viral load on ART (median, IQR)** | 124 (124-10,430) | 24,629 | 95.2% | 124 (46-164) | 7,567 | 54.0% |
| **Log10 (max viral load) (median, IQR)** | 2.1 (2.1- 4.1) | 23,722 | 91.7% | 2.1 (2.1- 2.6) | 6,000 | 42.8% |
| **Maximum viral load on ART >1000 (*n*, %)** | 7,800 (31.7%) | 24,629 | 95.2% | 1,312 (17.3%) | 7,567 | 54.0% |
| **Most recent viral load on ART as of 31 Dec 2014 (median, IQR)** | 124 (84-124) | 15,472 | 59.8% | 124 (0-124) | 6,779 | 48.4% |
| **Log10 (most recent viral load on ART) (median, IQR)** | 2.1 (2.1-2.3) | 12,515 | 48.4% | 2.1 (2.1- 2.2) | 4,834 | 34.5% |
| **Most recent viral load on ART >1000 (*n*, %)** | 2250 (14.5%) | 15,472 | 59.8% | 745 (11.0%) | 6,779 | 48.4% |
| **Achieved viral suppression on ART <400 (*n*, %)** | 22,431 (95.0%) | 23,625 | 91.3% | 4,781 (89.2%) | 5,361 | 38.2% |
| **Pregnancy at ART initiation, women (*n*, %)** | 1,443 (8.0%) | 18,048 | 99.4% | 2,342 (23.4%) | 10,013 | 99.7% |
| **TB treatment at ART initiation (*n*, %)** | 6,092 (23.7%) | 25,718 | 99.4% | 2,501 (17.9%) | 13,965 | 99.6% |
| **Ever transferred within Khayelitsha (*n*, %)** | 1,533 (5.9%) | 25,864 | 100.0% | 153 (1.1%) | 14,020 | 100.0% |
| **Transferred into ART care (*n*,%)** | 3,258 (12.6%) | 25,864 | 100.0% | 1,185 (8.5%) | 14,020 | 100.0% |
| **Combined transfer (combines two variables above) (*n*,%)** | 4,272 (16.5%) | 25,864 | 100.0% | 1,289 (9.2%) | 14,020 | 100.0% |
| **ART adherence club membership, ever (*n*, %)** | 6,182 (35.4%) | 17,463 | 67.5% | 227 (2.8%) | 8,197 | 58.5% |
| **Weight at ART baseline, kg (median, IQR)** | 62.6 (54.5-72.7) | 16,079 | 62.2% | 67 (58- 79) | 7,707 | 55.0% |
| **Baseline ART regimen drug 1 (*n*, %)** | TDF 11,740 (45.4%)) | 25,864 | 100.0% | TDF 12,717 (90.7%) | 14,020 | 100.0% |
|  | d4T 8,569 (33.1%) |  |  | d4T 177 (1.3%) |  |  |
|  | AZT 3,477 (13.4%) |  |  | AZT 300 (2.1%) |  |  |
|  | ABC 80 (0.3%) |  |  | ABC 39 (0.3%) |  |  |
|  | missing 1,998 (7.7%) |  |  | missing 787 (5.6%) |  |  |
| **Baseline ART regimen drug 3 (*n*, %)** | EFV 16,978 (65.6%) | 25,864 | 100.0% | EFV 12,994 (92.7%) | 14,020 | 100.0% |
|  | NVP 6,580 (25.4%) |  |  | NVP 118 (0.8%) |  |  |
|  | LPV/r 180 (0.7%) |  |  | LPV/r 76 (0.5%) |  |  |
|  | other 10 (0.04%) |  |  | other 10 (0.07%) |  |  |
|  | missing 2,116 (8.2%) |  |  | missing 822 (5.9%) |  |  |
| **ART regimen drug 1 at 1 Jan 2013 (*n*, %)** | TDF 14,646 (56.6%) | 25,864 | 100.0% | - | - | - |
|  | AZT 4,749 (18.4%) |  |  | - | - | - |
|  | d4T 3,503 (13.5%) |  |  | - | - | - |
|  | ABC 62 (0.2%) |  |  | - | - | - |
|  | missing 2,904 (11.2%) |  |  | - | - | - |
| **ART regimen drug 3 at 1 Jan 2013 (*n*, %)** | EFV 16,060 (62.1%) | 25,864 | 100.0% | - | - | - |
|  | NVP 4,906 (19.0%) |  |  | - | - | - |
|  | LPV/r 2,109 (8.2%) |  |  | - | - | - |
|  | other 13 (<0.1%) |  |  | - | - | - |
|  | missing 2,776 (10.7%) |  |  | - | - | - |
| **Most recent ART regimen drug 1 as of 31 Dec 2014 (*n*, %)** | TDF 18,773 (72.6%) | 25,864 | 100.0% | TDF 13,279 (94.7%) | 14,020 | 100.0% |
|  | AZT 4,812 (18.6%) |  |  | AZT 362 (2.6%) |  |  |
|  | d4T 1,725 (6.7%) |  |  | d4T 199 (1.4%) |  |  |
|  | ABC 59 (0.2%) |  |  | ABC 33 (0.2%) |  |  |
|  | missing 495 (1.9%) |  |  | missing 147 (1.1%) |  |  |
| **Most recent ART regimen drug 3 as of 31 Dec 2014 (*n*, %)** | EFV 18,729 (72.4%) | 25,864 | 100.0% | EFV 13,519 (96.4%) | 14,020 | 100.0% |
|  | NVP 3,474 (13.4%) |  |  | LPV/r 212 (1.5%)) |  |  |
|  | LPV/r 3,258 (12.6%) |  |  | NVP 126 (0.9%) |  |  |
|  | other 109 (0.4%) |  |  | other 17 (0.1%) |  |  |
|  | missing 294 (1.1%) |  |  | missing 146 (1.0%) |  |  |
| **Continuously in care (*n*, %) (no gap in care, no disengagement)** | 16,982 (65.7%) | 25,864 | 100.0% | 10,224 (72.9%) | 14,024 | 100.0% |
| **Previous gap in care of >180 days prior to 1 Jan 2013 (*n*, %)** | 3,677 (14.2%) | 25,864 | 100.0% | - | - | - |
| **Out of care at study entry (gap of 180 days includes 1 Jan 2013) (*n*, %)** | 4,244 (16.4%) | 25,864 | 100.0% | - | - | - |
| **Outcomes as of 31 Dec 2014** |  |  |  |  |  |  |
| **Alive and in care (*n*, %)** | 18,933 (73.2%) | 25,864 | 100.0% | 9,136 (65.2%) | 14,020 | 100.0% |
| **Dead (*n*, %)** | 369 (1.4%) |  |  | 223 (1.6%) |  |  |
| **Disengagement (*n*, %)** | 5,205 (20.1%) |  |  | 3,800 (27.1%) |  |  |
| **Transfer (*n*, %)** | 778 (3.0%) |  |  | 453 (3.2%) |  |  |
| **Silent transfer (*n*, %)** | 579 (2.2%) |  |  | 408 (2.9%) |  |  |

** Variables/data available at provincial clinics only*

*ABC, abacavir AZT, zidovudine; d4T, stavudine; EFV, efavirenz; LPV/r, lopinavir/ritonavir; NVP, nevirapine; TDF, tenofovir*
