# Supplementary material for: Contemporary disengagement from antiretroviral therapy in Khayelitsha, South Africa: A cohort study
Source: PLoS Med. 2017 Nov 7;14(11):e1002407. doi: 10.1371/journal.pmed.1002407 (PMC5675399; doi:10.1371/journal.pmed.1002407)
Supplement: S4 Table — *Complete case analysis included missing data from the “adherence club” variable, as this variable was only included in the provincial databases. Excluding the missing data from this variable would have eliminated all municipal patients from the model. This model did not contain imputed data. **This model contained imputed data but was restricted to those patients with national identification numbers to allow for accurate mortality ascertainment. ABC, abacavir; ART, antiretroviral therapy; AZT, zidovudine; CI, confidence interval; d4T, stavudine EFV, efavirenz; LPV/r, lopinavir/ritonavir; NVP, nevirapine; ref, reference; TB, tuberculosis; TDF, tenofovir; yrs, years. (DOCX) [file pmed.1002407.s011.docx]

**S4 Table: Cox Proportional Hazard model sensitivity analyses**

|  | **Complete case analysis (*n*=21,716) *** | | **National identification numbers only (imputed) (*n*=21,216) **** | |
| --- | --- | --- | --- | --- |
| **Variable** | **Hazard Ratio** | **95% CI** | **Hazard Ratio** | **95% CI** |
| **Age category** | | | | |
| *10-20 years* | 1.32 | 1.09 - 1.61 | 2.06 | 1.77 - 2.40 |
| *20-30 yrs* | 1.54 | 1.42 - 1.66 | 1.47 | 1.38 - 1.57 |
| *30-40 yrs* | ref | ref | ref | ref |
| *40-50 yrs* | 0.91 | 0.84 - 1.00 | 0.93 | 0.86 - 1.01 |
| *50-60 yrs* | 0.84 | 0.73 - 0.97 | 0.9 | 0.78 - 1.02 |
| *>60 yrs* | 0.91 | 0.68- 1.22 | 1.09 | 0.84 - 1.41 |
| **Sex / pregnancy** | | | | |
| *Non-pregnant women* | ref | ref | ref | ref |
| *Pregnant women* | 1.61 | 1.44 - 1.80 | 1.51 | 1.39 - 1.64 |
| *Men* | 1.08 | 1.00 - 1.16 | 1.14 | 1.07 - 1.22 |
| **TB treatment at ART initiation** | 1.19 | 1.10 - 1.28 | strata | strata |
| **Any transfer** | 0.86 | 0.78 - 0.94 | strata | strata |
| **Previous gap in care of >180 days** | 1.51 | 1.39 - 1.65 | strata | strata |
| **Provincial (vs. City) clinic** | 1.23 | 1.15 - 1.32 | 0.86 | 0.81 - 0.92 |
| **Baseline CD4 (cells/μl)** | | | | |
| *>350* | ref | ref | ref | ref |
| *200-350* | 0.67 | 0.59 - 0.76 | 0.57 | 0.52 - 0.63 |
| *50-200* | 0.56 | 0.49 - 0.64 | 0.44 | 0.40 - 0.49 |
| *<50* | 0.49 | 0.42- 0.57 | 0.36 | 0.31 - 0.41 |
| **Most recent CD4 as of 31 Dec 2014 (cells/μl)** |  |  |  |  |
| *>350* | ref | ref | ref | ref |
| *200-350* | 1.97 | 1.82- 2.13 | 1.98 | 1.83 - 2.14 |
| *50-200* | 2.99 | 2.70 - 3.30 | 2.98 | 2.68 - 3.32 |
| *<50* | 3.02 | 2.53 - 3.61 | 2.84 | 2.35 - 3.44 |
| **Viral load suppressed ever** | 0.5 | 0.46 - 0.56 | 0.6 | 0.54 - 0.67 |
| **ART adherence club membership, ever** | 0.3 | 0.26 - 0.34 | 0.28 | 0.25 - 0.33 |
| *missing* | 1 (omitted) | omitted | - | - |
| **Most recent ART regimen drug 1 as of 31 Dec 2014** |  |  |  |  |
| *Other* | ref | ref | ref | ref |
| *d4T* | 1.83 | 1.63 - 2.06 | 1.57 | 1.38 - 1.79 |
| **Most recent ART regimen drug 3 as of 31 Dec 2014** |  |  |  |  |
| *EFV* | ref | ref | ref | ref |
| *NVP* | 1.2 | 1.09 - 1.34 | 1.13 | 1.01 - 1.27 |
| *LPV/r* | 0.73 | 0.66 - 0.81 | 0.66 | 0.59 - 0.74 |
| *Other* | 0.21 | 0.09 - 0.51 | 0.21 | 0.10 - 0.45 |

**Complete case analysis included missing data from the “adherence club” variable, as this variable was only included in the provincial databases. Excluding the missing data from this variable would have eliminated all “City” patients from the model. This model did not contain imputed data.*

***This model contained imputed data but was restricted to those patients with national identification numbers to allow for accurate mortality ascertainment.*

*ABC, abacavir; ART, antiretroviral therapy; AZT, zidovudine; CI, confidence interval; d4T, stavudine EFV, efavirenz; LPV/r, lopinavir/ritonavir; NVP, nevirapine; ref, reference; TB, tuberculosis; TDF, tenofovir; yrs, years*
