# Supplementary material for: Contemporary disengagement from antiretroviral therapy in Khayelitsha, South Africa: A cohort study
Source: PLoS Med. 2017 Nov 7;14(11):e1002407. doi: 10.1371/journal.pmed.1002407 (PMC5675399; doi:10.1371/journal.pmed.1002407)
Supplement: S5 Table — *Different from initial outcomes for those who disengaged as presented in Table 3. (DOCX) [file pmed.1002407.s012.docx]

**S5 Table. Overall final outcomes* for silent transfers and those who disengaged, as locally ascertained by Western Cape province data systems and the National Death Registry**

| **MORTALITY** | **Total *n*** | **Deaths** | **%** |
| --- | --- | --- | --- |
| Patients who disengaged + silent transfers: first outcome = death | 9,992 | 67 | 0.7% |
| Patients who disengaged: first outcome = death | 9,005 | 67 | 0.7% |
| Patients who disengaged + silent transfers: total death | 9,992 | 262 | 2.6% |
| Patients who disengaged: total death | 9,005 | 237 | 2.6% |
| Patients who disengaged + silent transfers with national identification numbers: first outcome = death | 6,221 | 58 | 0.9% |
| Patients who disengaged with national identification numbers: first outcome = death | 5,463 | 58 | 1.1% |
| Patients who disengaged + silent transfers with national identification numbers: total death | 6,221 | 237 | 3.8% |
| Patients who disengaged with national identification numbers: total death | 5,463 | 213 | 3.9% |
| **HOSPITALIZATIONS** | **Total *n*** | **Hospitalizations** | **%** |
| Disengaged + silent transfers | 9,992 | 1,636 | 16.4% |
| Disengaged only | 9,005 | 1,459 | 16.2% |

**Different from initial outcomes for those who disengaged as presented in Table 3*
