# Supplementary material for: Contemporary disengagement from antiretroviral therapy in Khayelitsha, South Africa: A cohort study
Source: PLoS Med. 2017 Nov 7;14(11):e1002407. doi: 10.1371/journal.pmed.1002407 (PMC5675399; doi:10.1371/journal.pmed.1002407)
Supplement: S6 Table — * Variables/data available at provincial clinics only. ABC, abacavir; AZT, zidovudine; d4T, stavudine; EFV, efavirenz; LPV/r, lopinavir/ritonavir; NVP, nevirapine; TB, tuberculosis; TDF, tenofovir. (DOCX) [file pmed.1002407.s013.docx]

**S6 Table: Patient characteristics for those who disengaged and returned to care vs. failed to return**

| **Variable** | **Patients who returned to care** | | | **Patients who failed to return** | | |
| --- | --- | --- | --- | --- | --- | --- |
| # of participants (*n*) | 2,976 | # of patients with complete data | % complete data | 4,191 | # of patients with complete data | % complete data |
| Age at 1 January 2013, years (median, IQR) | 32.6 (27.1 - 39.3) | 2,976 | 100.0% | 32.2 (26.7 - 39.4) | 4,191 | 100.0% |
| Male sex (*n*, %) | 975 (32.8%) | 2,976 | 100.0% | 1,422 (33.9%) | 4,191 | 100.0% |
| Months on ART at 31 Dec 2014 (median, IQR) | 19 (5.7 - 42.2) | 2,976 | 100.0% | 16.2 (5.1 - 41.3) | 4,191 | 100.0% |
| Median year of starting ART (median, IQR) | 2012 (2010 - 2013) | 2,976 | 100.0% | 2012 (2010 - 2013) | 4,191 | 100.0% |
| Baseline CD4 count, cells/μL (median, IQR) | 186 (106 - 276) | 2,585 | 86.9% | 207 (122 - 303) | 3,581 | 85.4% |
| Most recent CD4 count as of 31 Dec 2014, cells/μL (median, IQR) | 318 (192 - 482) | 2,599 | 87.3% | 334 (211 - 510) | 3,638 | 86.8% |
| Most recent viral load >1000 on ART as of 31 Dec 2014 (*n*, %) | 437 (24.5%) | 1,787 | 60.0% | 469 (19.6%) | 2,390 | 57.0% |
| Achieved viral suppression on ART (<400) (*n*, %) | 1,609 (85.6%) | 1,879 | 63.1% | 2,180 (88.1%) | 2,475 | 59.1% |
| Initiated ART during pregnancy (women only) (*n*, %) | 280 (14.1%) | 1,991 | 99.5% | 531 (19.3%) | 2,755 | 99.5% |
| TB treatment at ART initiation (*n*, %) | 685 (23.1%) | 2,965 | 99.6% | 902 (21.6%) | 4,172 | 99.5% |
| Ever transferred within Khayelitsha (*n*, %) | 136 (4.6%) | 2,976 | 100.0% | 151 (3.6%) | 4,191 | 100.0% |
| Transferred into ART care (*n*,%) | 403 (13.5%) | 2,976 | 100.0% | 460 (11.0%) | 4,191 | 100.0% |
| ART club membership, ever (*n*, %) * | 137 (7.4%) | 1,855 | 62.3% | 209 (8.2%) | 2,547 | 60.8% |
| Weight at ART baseline in kg (median, IQR) * | 62.5 (54.6 - 72.4) | 1,671 | 56.1% | 63.1 (55.2 - 73.5) | 2,334 | 55.7% |
| Baseline ART regimen drug 1 (*n*, %) | TDF 2,010 (67.5%) | 2,976 | 100.0% | TDF 2,919 (69.7%) | 4,191 | 100.0% |
|  | d4T 513 (17.2%) |  |  | d4T 653 (15.6%) |  |  |
|  | AZT 233 (7.8%) |  |  | AZT 281 (6.7%) |  |  |
|  | ABC 6 (0.2%) |  |  | ABC 12 (0.3%) |  |  |
|  | missing 214 (7.2%) |  |  | missing 326 (7.8%) |  |  |
| Baseline ART regimen drug 3 (*n*, %) | EFV 2,299 (77.3%) | 2,976 | 100.0% | EFV 3,344 (79.8%) | 4,191 | 100.0% |
|  | NVP 430 (14.5%) |  |  | NVP 471 (11.2%) |  |  |
|  | LPV/r 17 (0.6%) |  |  | LPV/r 25 (0.6%) |  |  |
|  | Other 1 (0.03%) |  |  | Other 4 (0.1%) |  |  |
|  | missing 229 (7.7%) |  |  | missing 347 (8.3%) |  |  |
| Most recent ART regimen drug 1 as of 31 Dec 2014 (*n*, %) | TDF 2,377 (79.9%) | 2,976 | 100.0% | TDF 3,505 (83.6%) | 4,191 | 100.0% |
|  | d4T 200 (6.7%) |  |  | d4T 234 (5.6%) |  |  |
|  | AZT 346 (11.6%) |  |  | AZT 393 (9.4%) |  |  |
|  | ABC 4 (0.13%) |  |  | ABC 10 (0.2%) |  |  |
|  | missing 49 (1.7%) |  |  | missing 49 (1.2%) |  |  |
| Most recent ART regimen drug 3 as of 31 Dec 2014 (*n*, %) | EFV 2,425 (81.5%) | 2,976 | 100.0% | EFV 3,562 (85.0%) | 4,191 | 100.0% |
|  | NVP 269 (9.0%) |  |  | NVP 279 (6.7%) |  |  |
|  | LPV/r 242 (8.1%) |  |  | LPV/r 305 (7.3%) |  |  |
|  | Other 3 (0.1%) |  |  | Other 4 (0.1%) |  |  |
|  | missing 37 (1.2%) |  |  | missing 41 (1.0%) |  |  |
| Previous gap in care of >180 days (*n*, %) | 643 (21.6%) | 2,976 | 100.0% | 752 (17.9%) | 4191 | 100.0% |

*Variables/data available at provincial clinics only

*ABC, abacavir; AZT, zidovudine; d4T, stavudine; EFV, efavirenz; LPV/r, lopinavir/ritonavir; NVP, nevirapine; TB, tuberculosis; TDF, tenofovir*
