# Supplementary material for: Contemporary disengagement from antiretroviral therapy in Khayelitsha, South Africa: A cohort study
Source: PLoS Med. 2017 Nov 7;14(11):e1002407. doi: 10.1371/journal.pmed.1002407 (PMC5675399; doi:10.1371/journal.pmed.1002407)
Supplement: S7 Table — *Those who returned to care included all patients who returned to a clinic in the Western Cape ≥180 days after their last visit. Those who did not return to care included patients who did not have any follow-up information found in the Western Cape databases. We excluded silent transfers, and patients who had disengaged but whose first data point after disengagement was either a) death or b) hospital contact. **Variables selected for this model were the same variables selected for the multivariable Cox model in Table 2; however, CD4 variables were refined to more closely investigate a potential trend. ART, antiretroviral therapy; CI, confidence interval; d4T, stavudine; EFV, efavirenz; LPV/r, lopinavir/ritonavir; NVP, nevirapine; ref, reference; TB, tuberculosis; yrs, years. (DOCX) [file pmed.1002407.s014.docx]

**S7 Table: Logistic regression model for odds of not returning to care, for those who disengaged (n=7,167)***

| **Variable**** | **Odds ratio** | **95% CI** |
| --- | --- | --- |
| **Age category** | | |
| *10-20 years* | 1.08 | 0.84 - 1.38 |
| *20-30 yrs* | 1.09 | 0.97 - 1.23 |
| *30-40 yrs* | ref | ref |
| *40-50 yrs* | 1.01 | 0.88 - 1.16 |
| *50-60 yrs* | 1.19 | 0.95 - 1.49 |
| *>60 yrs* | 1.76 | 1.11 - 2.78 |
| **Sex/pregnancy** | | |
| *Nonpregnant women* | ref | ref |
| *Pregnant at ART initiation (women)* | 1.36 | 1.15 - 1.61 |
| *Men* | 1.16 | 1.04 - 1.29 |
| **Time on ART until disengagement (months)** | 1.01 | 1.00 - 1.01 |
| **TB treatment at ART initiation** | 0.92 | 0.82 - 1.04 |
| **Any transfer** | 0.73 | 0.64 - 0.84 |
| **Previous gap in care of >180 days** | 0.83 | 0.72 - 0.96 |
| **Provincial clinic** | 0.89 | 0.80 - 0.99 |
| **Baseline CD4 (cells/μL)** | | |
| *>500* | ref | ref |
| *350-500* | 1.16 | 0.82 - 1.64 |
| *200-350* | 0.79 | 0.59 - 1.05 |
| *100-200* | 0.68 | 0.50 - 0.94 |
| *50-100* | 0.6 | 0.41 - 0.89 |
| *25-50* | 0.67 | 0.45 - 1.02 |
| *<25* | 0.67 | 0.43 - 1.04 |
| **Most recent CD4 as of 31 Dec 2014 (cells/μL)** | | |
| *>500* | ref | ref |
| *350-500* | 0.85 | 0.72 - 1.02 |
| *200-350* | 0.97 | 0.82 - 1.14 |
| *100-200* | 0.97 | 0.78 - 1.20 |
| *50-100* | 1.02 | 0.73 - 1.42 |
| *25-50* | 1.02 | 0.63 - 1.66 |
| *<25* | 1.01 | 0.59 - 1.72 |
| **Viral load undetectable ever during ART** | 1.09 | 0.93 - 1.28 |
| **ART adherence club membership, ever** | 1.12 | 0.87 - 1.43 |
| **Most recent ART regimen drug 1 as of 31 Dec 2014** |  |  |
| *Other* | ref | ref |
| *d4T* | 0.83 | 0.67 - 1.02 |
| **Most recent ART regimen drug 3 as of 31 Dec 2014** |  |  |
| *EFV* | ref | ref |
| *NVP* | 0.71 | 0.58 - 0.86 |
| *LPV/r* | 0.88 | 0.72 - 1.07 |
| *Other* | 0.95 | 0.21 - 4.36 |

**Those who returned to care included all patients who returned to a clinic in the Western Cape >=180 days after their last visit. Those who did not return to care included patients who did not have any follow-up information found in the Western Cape databases. We excluded silent transfers, and patients who had disengaged but whose first data point after disengagement was either a) death or b) hospital contact.*

***Variables selected for this model were the same variables selected for the multivariable Cox model in Table 2; however, CD4 variables were refined to more closely investigate a potential trend.*

*ART, antiretroviral therapy; CI, confidence interval; d4T, stavudine; EFV, efavirenz; LPV/r, lopinavir/ritonavir; NVP, nevirapine; ref, reference; TB, tuberculosis; yrs, years*
